# Density Functional Studies on the Atomistic Structure and Properties of Iron Oxides: A Parametric Study

**DOI:** 10.3390/ma15238316

**Published:** 2022-11-23

**Authors:** Shujie Zhang, Kejiang Li, Yan Ma, Feng Guo, Chunhe Jiang, Zeng Liang, Yushan Bu, Jianliang Zhang

**Affiliations:** 1 School of Metallurgical and Ecological Engineering, University of Science and Technology Beijing, Beijing 100083, China; 2 Max−Planck−Institut für Eisenforschung, Max−Planck−Straße 1, 40237 Düsseldorf, Germany; 3 School of Physical Science and Information Technology, Liaocheng University, Liaocheng 252000, China

**Keywords:** iron oxides, density functional theory, atomic structure, parameter testing

## Abstract

With the aim to find the best simulation routine to accurately predict the ground−state structures and properties of iron oxides (hematite, magnetite, and wustite) using density functional theory (DFT) with Hubbard-U correction, a significant amount of DFT calculations were conducted to investigate the influence of various simulation parameters (energy cutoff, K-point, U value, magnetization setting, smearing value, etc.) and pseudopotentials on the structures and properties of iron oxides. With optimized simulation parameters, the obtained equation of state, lattice constant, bulk moduli, and band gap is much closer to the experimental values compared with previous studies. Due to the strong coupling between the 2p orbital of O and the 3d orbital of Fe, it was found that Hubbard-U correction obviously improved the results for all three kinds of iron oxides including magnetite which has not yet been tested with U correction before, but the U value should be different for different oxides (3 ev, 4 ev, 4 ev for hematite, magnetite, and wustite, respectively). Two kinds of spin magnetism settings for FeO are considered, which should be chosen according to different calculation purposes. The detailed relationship between the parameter settings and the atomic structures and properties were analyzed, and the general principles for future DFT calculation of iron oxides were provided.

## 1. Introduction

Iron oxide (mainly including hematite, magnetite, and wustite) is a kind of common compound that exists widely in nature [1]. It has become the focus of many studies because of its diverse crystal forms and mutual transformation, which finally leads to obviously different properties. The study of the structures and properties of iron oxides in an atomistic scale is essentially important for a wide range of research fields, such as geology, mineralogy, biomedicine, physical chemistry, catalysis, and metallurgical industry [2,3,4].

Density functional theory (DFT) [5,6,7,8,9,10,11] has been widely used to simulate the structure and calculate the properties of iron oxides [12,13,14,15]. However, it is widely known that the strongly correlating d−electrons of transition metal form the valence band, resulting in incorrect structure and properties, especially underestimated band gap [16]. To address this problem, Hubbard-U correction is usually added to the DFT calculation [17,18,19], and a lot of calculations are required to obtain a suitable U value for each kind of oxide. Previous studies have shown that each material typically requires a semi−empirical U−value to accurately describe its electronic structures and properties [10,12,16,20,21,22,23,24].

The parametric settings in DFT calculations are essentially important to reproduce the experimental structure and properties accurately. Meng [20] et al. conducted DFT calculations for iron oxides with three density functional approximations, PBE, PBE+U, and Heyd-Scuseria-Ernzerhof screened hybrid functional (HSE), results indicated that each iron oxide has its most suitable parameters and pseudopotential to produce different properties accurately [9,20]. Besides, a lot of parameters including energy cutoff, K-point, smearing value, magnetization setting, etc. should be tested to achieve a good convergence and gain the most accurate ground state structure and energy, which require a significant amount of computation. Till now, we have not yet found any general rules or suggestions on how to set the parameters in the DFT calculation of iron oxides. To fill this gap, a quantitative comparison of iron oxide structures and properties by DFT while using different parameters and pseudopotentials should be performed so that the subsequent DFT calculation of iron oxides can be more conveniently, accurately, and computationally inexpensive.

In this paper, a significant amount of DFT calculations with Hubbard-U correction were conducted to investigate the influence of various simulation parameters (energy cutoff, K-point, U value, magnetization setting, smearing value, etc.) and pseudopotentials on the ground state structures and properties of iron oxides. Hubbard-U correction for oxygen was set for the first time in Fe_3_O_4_ calculations, and the unit cell parameters of the three oxides achieved better results. The effects of two kinds of FeO spin magnetic orientation settings [25,26] are also discussed. The equation of state (EOS) curves under different pseudopotentials with the optimized parameters are calculated and compared. The detailed influence of parameter setting is analyzed to provide a general rule for future iron oxide DFT calculation.

## 2. Simulation Methodology

**Figure 1:**
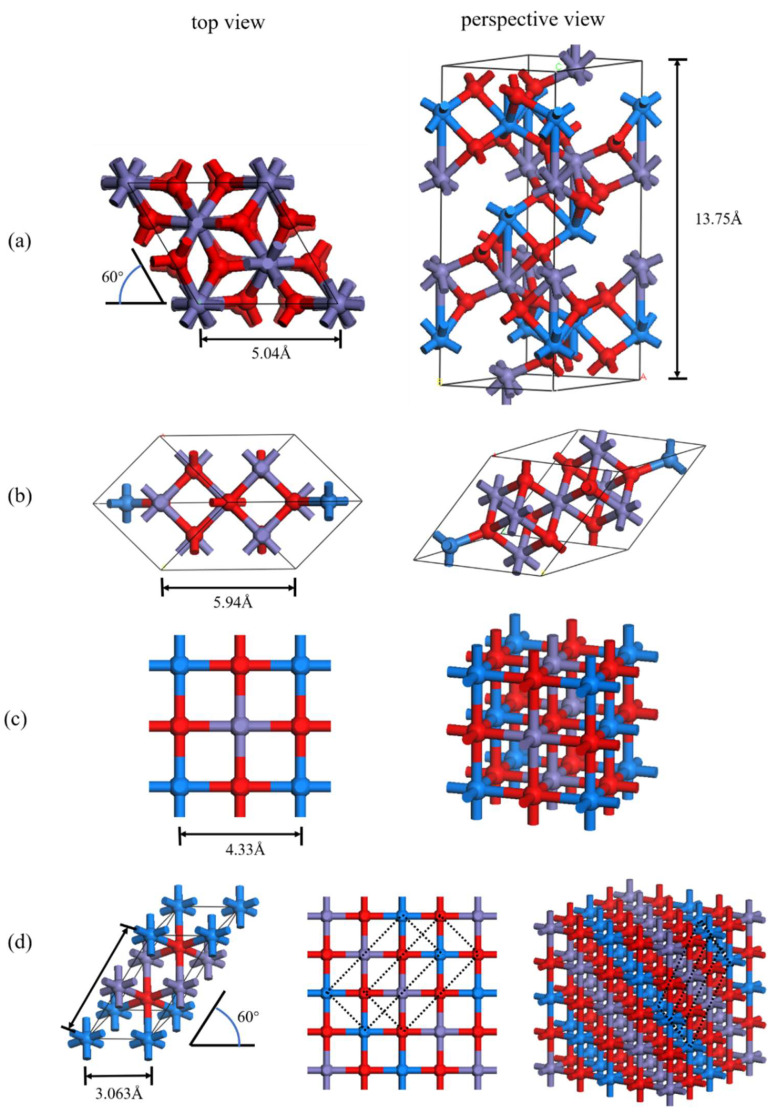
(**a**–**d**) are the unit cell models of Fe_2_O_3_, Fe_3_O_4_, FeO, and FeO*, respectively, with O atoms in red, Fe atoms in purple and blue (different colors indicate different spin directions). Left is top view; right is the perspective view. (**c**,**d**) are both FeO, but have different spin Settings.

All calculations in this paper have been applied as either the Perdew–Burke–Ernzerhof (PBE) version of the generalized gradient approximation (GGA) or the GGA + U method and a plane-wave-based DFT implemented in the open-sourced Quantum Espresso package was employed in all simulations [27,28]. For Fe_2_O_3_, the conventional unit cell is used for calculation, as shown in Figure 1a. Fe_3_O_4_ is calculated through the primitive cell to save the computation cost, as shown in Figure 1b. FeO is calculated by two kinds of spin settings, as shown in Figure 1c,d, respectively. One kind of setting was set layer by layer along the direction (100) (labeled with FeO), while the other kind of setting was set layer by layer along the direction (111) (labeled as FeO*). Conventional unit cell and 2 × 1 × 1 supercell (Figure 1d) were used for FeO and FeO*, respectively. Fe atoms with different spin directions are colored blue and purple respectively [21,29]. The variable cell relaxation module is used to calculate the relationship between parameters and unit cell parameters. Detailed cell parameters and atomic coordinates are shown in Tables S1–S3.

The main parameters are shown in Table 1 for calculation, and all the detailed parameters are shown in Tables S1–S3 in the supporting information. The detailed results of the parametric tests mentioned in the following results can be found in Tables S4–S45 in the supporting information. In the calculation, only the tested parameters change, and the other parameters remain unchanged.

## 3. Results

### 3.1. Parametric Study of Hematite

#### 3.1.1. The Parameters Influencing the Convergence

**Figure 2:**
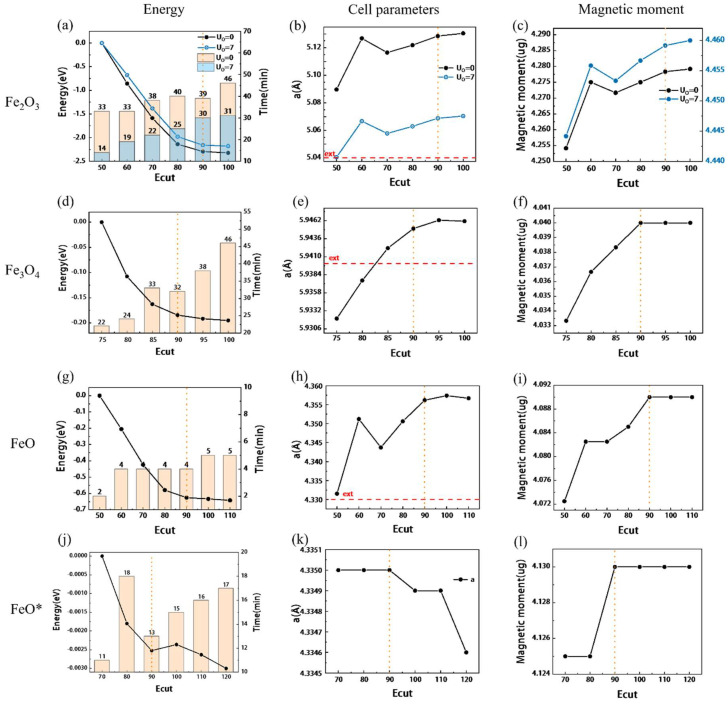
The test results of Ecut for three iron oxides, the horizontal direction is the parameters of the same oxide ((**a**–**c**) for Fe_2_O_3_, (**d**–**f**) for Fe_3_O_4_, (**d**–**i**) for FeO, and(**j**–**l**) for FeO*), and the vertical direction is the comparison of the same results. The histogram is the calculation time, the orange dotted line is the position of the final value, and the red line is the experimental value. The right coordinate axis in (**c**) corresponds to the blue line in the figure. FeO* is the result of the spin setting along (111).

**Figure 3:**
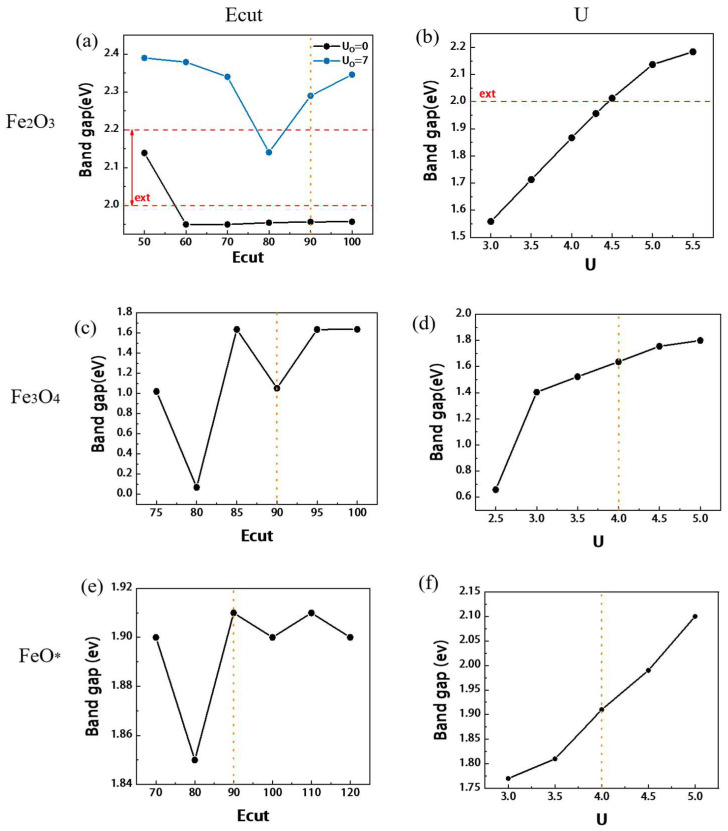
The results of the band gap for Fe_2_O_3_, Fe_3_O_4_, and FeO*, the horizontal direction is the same oxide parameter ((**a**,**b**) for Fe_2_O_3_, (**c**,**d**) for Fe_3_O_4_, (**e**,**f**) for FeO*), and the vertical direction is the result of the same influence parameter. The orange dotted line is the position of the final value, and the red line is the experimental value.

**Figure 4:**
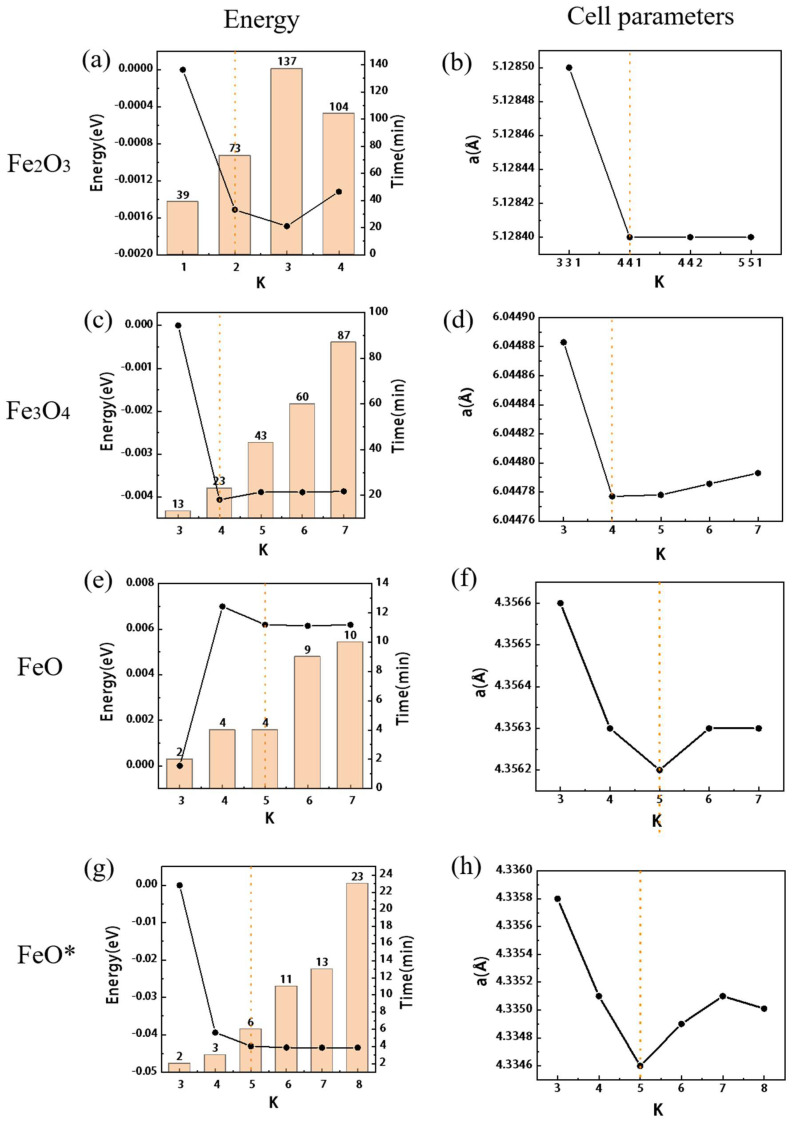
The test results of the k-point for three iron oxides, the horizontal direction is the parameter of the same oxide ((**a**,**b**) for Fe_2_O_3_, (**c**,**d**) for Fe_3_O_4_, (**e**,**f**) for FeO, (**g**,**h**) for FeO*), and the vertical direction is the comparison of the same calculation result. The histogram is the calculation time, the orange dotted line is the position of the final value.

The effect of energy cutoff (Ecut) is shown in Figure 2a–c. The change in the band gap is shown in Figure 3a. The yellow dotted line is the position of the final value, and the red line is the experimental value. The detailed results are shown in Table S5. A comparison between the experimental values [30,31,32] and reference [20] is made. To ensure the convergence of energy, lattice constant, and magnetic moment, the Ecut should be higher than 90 Ry. The calculation results with different k-point settings are shown in Figure 4a,b, and the detailed results are shown in Table S7. The influence of the k-point on energy, unit cell, band gap, etc. is very small. However, it has a significant effect on the calculation time. Compared with 4 × 4 × 1, the time of 4 × 4 × 2 is almost doubled, and the number of convergence calculations and energy are almost the same. Therefore, it is reasonable to choose 4 × 4 × 1 for hematite.

#### 3.1.2. The Effect of U Correction

**Figure 5:**
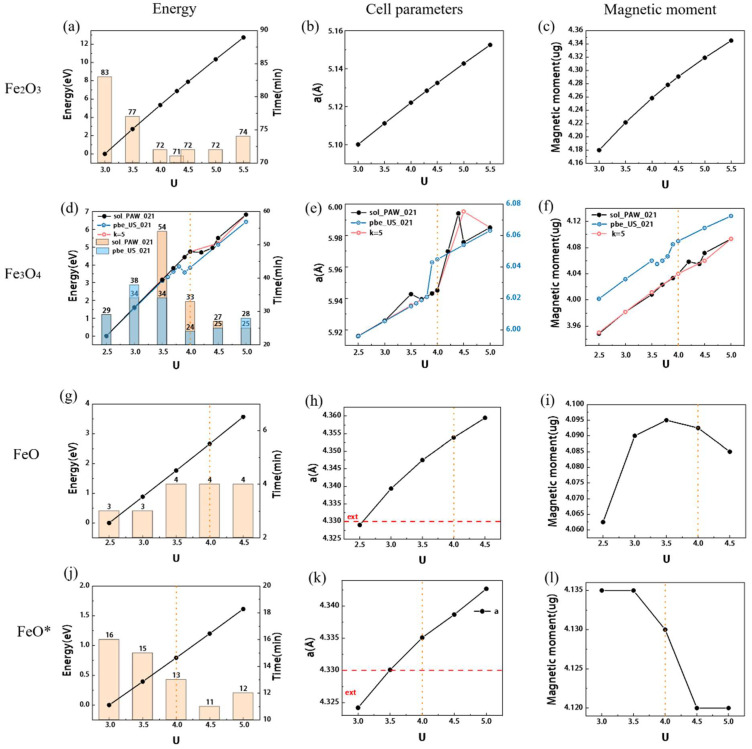
The test results for three iron oxides U, the horizontal direction is the parameters of the same oxide ((**a**–**c**) for Fe_2_O_3_, (**d**–**f**) for Fe_3_O_4_, (**g**–**i**) for FeO, (**j**–**l**) for FeO*), and the vertical direction is the comparison of the same results. The histogram is the calculation time, the orange dotted line is the position of the final value, and the red line is the experimental value.

The calculation results with different U_Fe_ are shown in Table S8. The effect of energy, unit cell parameters, and magnetic moment are shown in Figure 5a–c, and the band gap change is shown in Figure 3b. U_Fe_ has a significant effect on most of the properties. With the increase of U_Fe_, the unit cell parameters, magnetic moment and convergence energy will increase almost linearly. The addition of U also reduces the computation time, while increasing the band gap and magnetic moment closer to the experimental values. After adding U_O_, the band gap and magnetic moment will be larger, but the unit cell parameters will be relatively smaller, as shown in Figure 2b, which will be closer to the experimental value. Previous studies [33] have tested and interpreted the calculated values of U_O_ accordingly [34,35,36]. The setup of U_O_ allows to consistently handle small polarons of electrons and holes in hematite. In halides and oxides, charge excitations can couple to lattice modes, leading to the formation of polarons. It was found [35] that the generation of small polarons will increase the band gap maximum and can be better described by the calculation of U. In Fe_2_O_3_, small polarons of electrons and holes are more dominant than large polarons, and the unit cell parameters and magnetic moments are closer to the experimental values after adding U_O_. This also confirms the necessity of U_O_ in Fe_2_O_3_ calculations [37].

#### 3.1.3. Effect of Pseudopotential and Exchange Functional

To understand the influence of pseudopotentials, five pseudopotentials including both ultrasoft (US) and projector augmented wave (PAW) pseudopotentials were used, named in Table S4 for subsequent discussion. The results are presented in Table S10 and compared with previous calculations [20,38,39] and experimental values [30,31,32]. It was found that the lattice constant produced with pbe_US_021 is generally larger than that produced with the others. Under the same conditions, the other three sets of pseudopotentials are closer to the experimental values. The sol_PAW_021/100 will generally converge more slowly, and the band gap will be lower than the other two groups. In the result, sol_US_021 was also tested under the same conditions, but the calculation showed that the self-consistent field (SCF) did not converge within 100 steps. The band gap of pbe_US_MIT is larger when other parameters are the same.

There are some parameters including degauss, starting magnetization, mixing factor for self-consistency, and convergence threshold for self-consistency, which have little effect on the structure and properties, but influence the convergence speed, and the results are shown in Tables S11–S15.

### 3.2. Parametric Study of Magnetite

#### 3.2.1. Effect of K-Point and Pseudopotential

Since the selection of k-points has a great influence on calculation time, k-points are calculated first while keeping a high Ecut value. The results are shown in Table S16, as shown in Figure 4c,d. Considering the convergence and calculation time, 4 × 4 × 4 is the best K-point choice. We further investigate the influence of different pseudopotentials and compared them with the previous calculations [40,41,42] and experimental values [43,44,45]. The results are shown in Table S17. The calculation convergence speed of pbe_US_MIT is the fastest, but to ensure the accuracy of the lattice constant, sol_PAW_021 has a great advantage and is the closest to the actual value of the experiment. The computation time using sol_PAW_100 is almost double that of other pseudopotential sets, which is much longer than sol_PAW_021. Therefore, sol_PAW_021 is temporarily used for subsequent calculations.

#### 3.2.2. Effect of Energy Cutoff and U_Fe_

The test results with changing Ecut are shown in Figure 2d–f and Figure 3c, and the detailed results are shown in Table S18. The energy tends to converge when Ecut is above 90 Ry, and the band gap and magnetic moment tend to be stable when it is 90–95 Ry. Therefore, it is necessary to take at least 90 Ry, and continue to use 90 Ry for subsequent calculations. The effect of U_Fe_ is shown below in Figure 3d and the black line in Figure 5d–f. All detailed results are shown in Table S19. Consistent with the principle for Fe_2_O_3_, with the increase of U_Fe_, the unit cell parameters, band gap, magnetic moment, and convergence energy will increase. However, it does not have a completely linear growth trend, which is different from hematite. In the range of 4.0–4.5 eV, the energy and magnetic moment showed an arc-shaped curve that first decreased and then increased, and the unit cell volume suddenly increased. When U = 4.4 eV, the computation time is almost twice as long as that of the others. After doing a series of statistics, it is found that since the unit cell uses the original cell of α = 60° when U = 4.4 eV, the calculation time is doubled due to the large deviation of 0.3° in the angle of the unit cell. The same situation also occurs when U = 3.5 eV, but the deviation is smaller by 0.06°.

To investigate the situation with different pseudopotentials, we use pbe_US_021 (blue line in Figure 5d–f) and use a k-point of 5 × 5 × 5 (pink line in Figure 5d–f). A simple calculation is also completed, and the detailed results are shown in Tables S20 and S21. The same result was obtained, but the fluctuation interval of U is different. In pbe_US_021, the angle change is not obvious, but the angle change of K point U = 4.5 eV of 5 × 5 × 5 is obvious, and the convergence time is also sharply prolonged. When the unit cell volume suddenly increases, the Fermi level will decrease as shown in Figure S1. It is considered that the breakpoint with the change of U value is caused by the different accuracy of cell angle calculation under different conditions. When the k-point becomes larger and the accuracy increases, the breakpoint will gradually become more regular. Similarly, the regularity is also found in the calculation of FeO as discussed in the next section.

There are also some parameters that have little effect on the results, and the calculation results are placed in Tables S22–S26 and Figure S2. 

### 3.3. Parametric Study of Wustite

In this chapter, the configuration of Figure 1c and the setting of spin magnetism are used for calculation. The difference between the two spin Settings is explained in Section 3.3.3.

#### 3.3.1. Effect of K-Point and Pseudopotential

K-point calculations were performed with high Ecut value and the results are shown in Table S27 and Figure 4e,f. Considering the convergence energy and computation time, 5 × 5 × 5 is the best K-point choice. In the calculation, it was found that the square unit cell of part of FeO is susceptible to elongation or shrinkage, which affects the convergence. So, the value of a/b was used in the table as a measure. Results with different pseudopotentials are shown in Table S28 and compared with the previous calculation results [11,46,47] and experimental values [48,49]. The unit cell parameters of pbe_US_MIT are more accurate, but the convergence time is longer. The unit cell produced with pbe_US_021 is slightly larger, but the aspect ratio (a/b) is suitable, and the convergence time is the shortest. The time, unit cell parameters, and even the convergence energy produced with sol_PAW_100 and sol_PAW_021 are not significantly different and are all slightly smaller, while the convergence results of the Sol_US_021 group are still poor. Subsequent calculations are calculated using pbe_US_021.

#### 3.3.2. Effect of Energy Cutoff and U_Fe_

**Figure 6:**
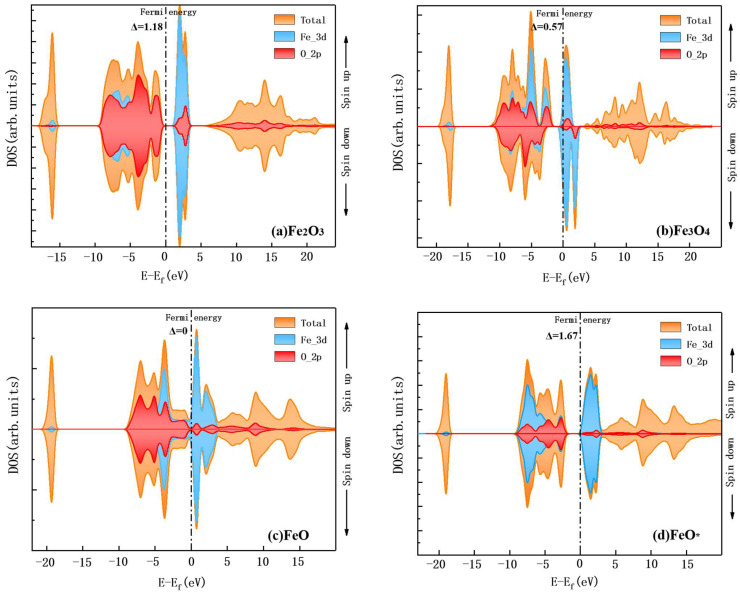
The test results for three iron oxides DOS. The total DOS is shown in orange, the Fe atom 3d orbital in blue, and the O atom 2p orbital in red.

The effect of Ecut is shown in Figure 2g–i, and the detailed results can be found in Table S29. The convergence energy and lattice parameters tend to be stable when Ecut is above 90 Ry. When calculating the band gap, it is found that the calculation result is almost 0 [11,50], as shown in Figure 6c, and the subsequent statistics are not carried out. The effect of U_Fe_ is shown in Figure 5g–i, and the detailed results are shown in Table S30. As U increases, the convergence energy and cells increase, and the Fermi level decreases accordingly, which can be used to adjust to the actual value. However, the unit cell parameters do not show a proportional increase, and the change in the magnetic moment is more obvious. When U = 4 eV, although the unit cell parameter will be slightly larger, the aspect ratio is basically 1, which is more in line with the unit cell situation, so U = 4 eV is preferred. There are also some parameters of FeO that have little effect on the results, and the results are placed in Tables S31–S36 and Figure S3.

#### 3.3.3. Effects of Different Spin Orientation Settings

The same calculation was performed along the setting of (111) spin magnetism using the configuration of Figure 1d, and the result is denoted as FeO*. The results are shown in Tables S37–S40, and Figure 2j–i, Figure 4f,g and Figure 5j–i. There are two significant differences between FeO* and FeO results. 1. With different cell deformation directions, FeO will stretch and shorten along the direction of (100), that is, a/b ≠ 1, while FeO* is along the rhombus direction, and the angle changes slightly. 2. The band gap of FeO is almost 0, but FeO* has a good band gap, and the variation law is very similar to hematite and magnetite, as shown in Figure 3e,f. According to Figure 6, we can see that Fe_3d dominates above the Fermi level, and Fe_3d and O_2p act together below the Fermi level. In FeO, the ratio of O_2p is overestimated so that the band gap is almost zero. This may be due to the effect of cell distortion caused by the different spin arrangements of FeO [27]. However, the spin direction of FeO* is not easy to set, especially if you want to use primitive cells or small surfaces, and FeO* requires longer computation time even for the same parameters. Therefore, it is suggested to use the spin magnetic setting along the (100) direction if the surface reaction calculation is carried out, and the calculation of the small system is not affected by the band gap. If used for semiconductor calculations, such as those that have a great deal to do with magnetic band gaps, the spin setup along (111) will give more accurate results.

There are also some parameters of FeO* that have little effect on the results, and the results are placed in Tables S41–S46. 

### 3.4. The Best Parameter Set for Iron Oxides

As shown in Table 2, the parameter Settings is considered to be the most appropriate in the test of this paper and the corresponding results.

## 4. Discussion

### 4.1. Effect of Pseudopotential

Local density approximation (LDA) was proposed earlier, it is also widely used in material science research. It is relatively accurate and generally gives a good estimate of the structure and elastic properties, but it overestimates the binding energy, underestimates the activation energy of the reaction, and excessively favors the high-spin structure, etc. Compared with LDA, GGA can calculate atomic and molecular energy more accurately, correct over binding, and obtain more accurate reaction activation energy. The GGA is not always superior to the LDA. The GGA function usually gives high lattice parameters, but from the results, this can be adjusted by the parameters. For iron oxides, the spin structure has a very important influence. We hope to continue the calculation on the surface, hoping to get more accurate activation energy in the follow-up study, which GGA is better at. Using Heyd-Scuseria-Ernzerhof screening mixed functional (HSE) calculation will combine the advantages of LDA and GGA, reduce some errors of GGA functional, and expand the band gap to match the experiment. In previous calculations, HSE (a = 0.15) was used as the best choice [20]. However, more accurate cell parameters are obtained in the results of this paper, and the setting of UO also makes the band gap of Fe_3_O_4_ more accurate.

A total of five groups of pseudopotentials were selected for calculation. From Tables S10, S17, S28 and S37, under the same parameters, pbe_US_021 tends to produce larger lattice parameters, while pbe_US_MIT will obtain higher magnetic moment results. Sol_paw_021/100 usually has a longer convergence time and similar results, but sol_PAW_021 is more accurate in terms of lattice constants, while sol_PAW_100 usually has a smaller unit cell structure and magnetic moment. pbe_US_MIT has a better convergence rate for both Fe_2_O_3_ and Fe_3_O_4_, but it is the most difficult one for FeO. It is thought that this may be due to the inaccurate calculation of cell shape, which leads to the difficulty of calculation. This can be seen by observing the results of pbe_US_MIT and SOL_PAW_021/011 calculations in FeO, both of which are slightly stretched or compressed, resulting in difficult convergence and increased computation time. Additionally, according to FeO*, the larger the change in the angle, the longer the convergence time. In summary, pbe_US_021 and pbe_US_MIT are more suitable choices. After parameter selection and optimization, the cell parameters have been very accurate, and even if the results are not the closest, the error is small. In the case of pbe_US_021 pseudopotential, the energy band and elastic modulus of Fe_2_O_3_ and Fe_3_O_4_ are greatly improved by the setting of the U_O_ parameter. The downside is that neither pbe_US_021 nor U_O_ Settings apply to FeO/FeO*. In contrast, pbe_US_MIT can calculate the cell parameters of all iron oxides more comprehensively but cannot accurately estimate the energy band.

### 4.2. The General Impact of U_Fe_

The U value can refer to the linear response method or the module using hp.x. The main purpose of this paper is to summarize the relationship between U, energy band, and cell parameters, provide a reference, and use this rule to choose the direction of predicting and modifying U values. Make sure you choose a value that describes the parameters you are interested in.

In order to overcome the error of the core calculation of the D state occupied by the transition metal oxides, the DFT + U method is proposed [57]. With the increase of U, the cell parameters, band gap, and magnetic moment almost increase linearly, which has a great influence on the results. This is because the method adds a Hubbard-like term to the total energy functional and uses the self-consistent field method to solve in the independent particle approximation (Equation (1) below), so the energy increases linearly with U. In Equation (1), n_I,α_ is the occupancy rate of α on the I [35] orbit.

(1) $$E_{DFT+U}=E_{DFT}+\frac{1}{2}\underset{I\propto }{\sum }U_{\propto }n_{I,\propto }\left(1-n_{I,\propto }\right)$$

However, not all the results are linear. The breakpoint appears in Fe_3_O_4_ in Figure 5d–f and when U = 4.4 eV in the FeO calculation process. In FeO, when point K changes from 4 × 4 × 4 to 5 × 5 × 5, the breakpoint becomes a downward curve (Figure 5I), and the magnetic moment no longer grows linearly. It is thought that this is due to the variation of cell parameters caused by the interatomic spin magnetic arrangement. In other words, in Fe_3_O_4_ and FeO, the change of generation parameters caused by U is the result of the joint influence of U, magnetic moment, and unit cell parameters. When K = 4 × 4 × 4, the FeO unit cell shows a critical junction change due to unstable convergence, which leads to a breakpoint in the result, but when K = 5 × 5 × 5, this change will be regular due to the more accurate calculation of the unit cell structure. In Fe_3_O_4_ (Figure 5d–f), it can also be seen that the breakpoints gradually decrease as the k-point increases. In a word, U_Fe_ = 4 can basically guarantee accuracy. If you need to try U_O_, U_O_ = 7–8 is the usual range.

### 4.3. General Influence of Other Parameters

The convergence energy decreases with the increase of Ecut and can basically converge when the three iron oxide parameters are set to 90–95 Ry, as shown in Figure 2. K-Point has very slight convergence, as shown in Figure 4, but if the convergence requirements are not met, unexplained non-convergence will be encountered in the subsequent parameter test. For example, Degauss found in the test that part of the results could not converge because the K-points were not dense enough, which would cause great interference in the test of the initial parameters. With the gradual increase of Gauss, the magnetic moment decreases rapidly after reaching a certain value, regardless of whether Gauss or Mv is selected for the seam as shown in Figures S2 and S3. It is perhaps for this reason that demagnetization is not too great for conductors. The initial magnetic moment, self-consistent mixing factor (mixing_beta), and self-consistent convergence threshold (conv_thr) have little effect on the results, but incorrect values may lead to convergence failure at the beginning of the test. According to the selection range of nbnd described in the manual, it is half the sum of the outer electrons, the metal needs to add 20%, but according to Tables S15, S24, S35, and S45, larger values are required. After stable convergence, the computation time will increase gradually.

### 4.4. Equation of State of Iron Oxides

**Figure 7:**
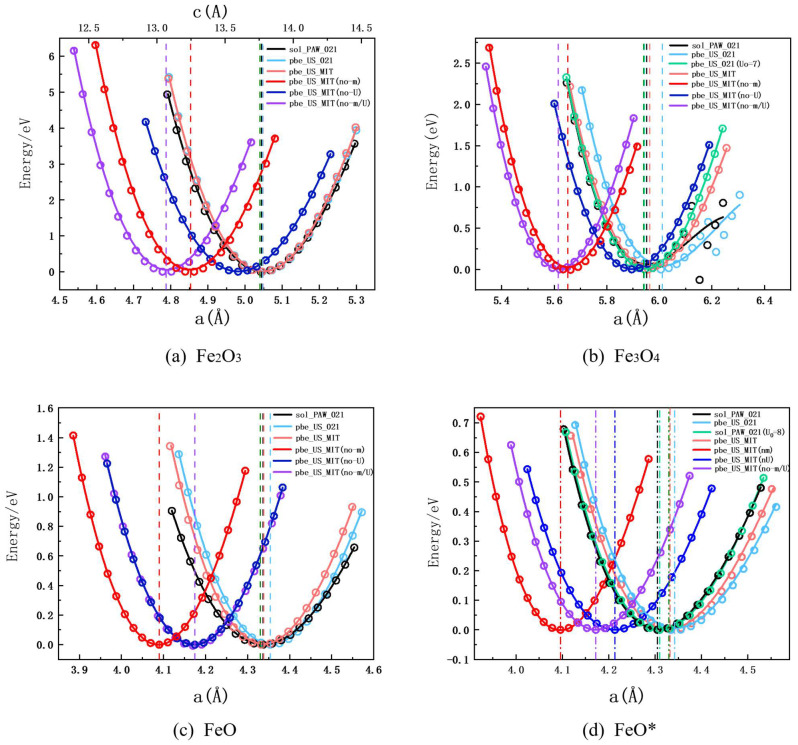
(**a**–**d**) are the equations of state of Fe_2_O_3_, Fe_3_O_4_, FeO, and FeO*, respectively. The circles represent the calculated data, and the lines corresponding to the colors are the fitting results by the Birch-Murnaghan equation of state. The dotted line is the V_O_ obtained by the corresponding color line, and the dark green dotted line is the experimental value.

The volumes, and corresponding energies of the three iron oxides (including FeO*) are calculated using the appropriate parameters. For the convenience of comparison, the energy in the figure is the difference between the energy corresponding to the calculated volume and the energy at the lowest point, and the abscissa is the unit cell parameter corresponding to the volume. By fitting the Birch-Murnaghan equation of state [58,59], the EOS curve in Figure 7 is obtained. The dashed lines in different colors are the result of V_0_ for different pseudopotentials and parameters (red, dark blue, and purple are the results without spin magnetism, without U, spin magnetism, and U off, respectively). The main parameters obtained from the fitting are shown in Table 3, where bold represents the result closest to the experimental value. Generally, the calculated properties agree very well with experimental results. The DOS calculation for each group of iron oxide EOS results closest to the experimental value is shown in Figure 6.

Compared with the experimental values, the calculated properties for Fe_2_O_3_ are the closest to the experimental results. This may be due to the U_O_ setting, so after testing (see Figure S4), a set of appropriate parameters was selected to carry out the Fe_3_O_4_ (U_O_ = 7) results, as shown in the light green line in Figure 7b and the data in Table 3. Compared with the same set of pseudopotentials without U_O_, the bad points caused by the expansion of unit cell parameters are repaired, and B_0_ is closer to the experimental value. At the same time, the band gap is 0.57, which is closer to the experimental value of 0.14 [55]. As shown in Figure 6, Fermi levels are not in the band gap, which is similar to the previous calculation [9,23,54], and when U_O_ = 0, Fermi levels are almost all in the band gap. This also means that the strong coupling between the 2p and Fe orbitals of O is often ignored in iron oxide calculations, resulting in inaccurate band gap calculations, and resulting in a bias in cell structure calculations, which U_O_ can repair. The same method has been tried in FeO and FeO* (see Figure S5), but no suitable U_O_ has been found so far, and U_O_ may not interact so strongly in wustite, or appropriate parameters have not been found for the time being. At the same time, B_0_ under two different spin Settings is compared, and there is not much difference. Therefore, it is considered that different Settings do not have much influence on the calculation of the overall cell volume energy. Therefore, if the calculation of the band gap is not considered, all can be selected.

In Figure 7, the red line, dark blue line, and purple line indicate the result with no spin magnetism setting, and results with both spin magnetism and U turned off, respectively. It can be seen from the comparison that the settings of spin and U have a great influence on the correct calculation of the energy-volume relationship of the unit cell. Without setting spin magnetism and U, both the unit cell parameters are greatly underestimated, and the bulk modulus is overestimated, especially without spin. From the position of the curve, the magnetic moment will have a greater effect on the unit cell parameters. Comparing the trends of the curves, it appears that the absence of U underestimates the effect of volume on energy, while the absence of magnetic moment overestimates. The shape and position of the purple line in Fe_3_O_4_ are closer to the red line, which indicates that spin magnetization has a greater effect on the unit cell parameters and bulk modulus of Fe_3_O_4_. In FeO, the purple line is almost consistent with the dark blue line, which indicates that the correct unit cell parameters of FeO require the combined effect of DFT+U and spin. When only U is used, the unit cell parameter is underestimated, and when only spin is set, there is no good effect. In FeO*, the purple line is distinguished from the dark blue line. The effect of the magnetic moment on the relationship between volume and energy is weaker. As with FeO, the unit parameter is greatly underestimated when only U is used.

## 5. Conclusions

Using density functional theory and Hubbard−U correction, the ground−state structures, and properties of hematite, magnetite, and wustite were systematically studied with different simulation parameters as well as pseudopotentials. The trade−off between accuracy and cost was achieved to predict the structure and properties of iron oxides close to the experimental results while keeping a relatively low computational cost. Even though there is no set of parameters that can be perfectly suitable to calculate all the properties for all three kinds of iron oxide, the best parameters and the general parametric selection rules for each oxide were obtained. It was validated that the strong coupling between the 2p orbital of O and the 3D orbital of Fe should be considered to produce the accurate structure and properties for each oxide, while the U value for each oxide is different. Two methods of setting FeO spin magnetism are considered, and the results are compared: the direction along (100) is more suitable for small systems and surface reactions, while the direction along (111) is more suitable for computing semiconductor systems. The presence or absence of spin magnetic moment and even the setting direction have a great influence on the unit cell parameters and bulk modulus. Some general rules are also summarized for important parameters influencing convergence and accuracy, which can be more convenient for future simulation research.

## Figures and Tables

**Table 1:** The main parameters and values used for testing.

	Hematite	Magnetite	Wustite(FeO/FeO*)
Ecut (Ry)	50, 60, 70, 80, 90, 100	75, 80, 90, 95, 100	50, 60, 70, 80, 90, 100, 110
Ecut (Ry) (U_O_ = 7)	50, 60, 70, 80, 90, 100	/	/
K-point	(3 3 1), (4 4 1), (4 4 2), (5 5 1)	3, 4, 5, 6, 7	3, 4, 5, 6, 7
U_Fe_ (eV)	3, 3.5, 4, 4.5, 5, 5.5	2.5, 3, 3.5, 4, 4.5, 5	2.5, 3, 3.5, 4, 4.5, 5
U_O_ (eV)	7.0	/	/

**Table 2:** The optimal parameters and results for the three iron oxides.

	Hematite (Tables S5–S15)	Magnetite (Tables S16–S26)	Wustite (FeO) (Tables S27–S36)	Wustite (FeO*) (Tables S37–S46)
Pseudopotential	pbe_US_MIT	sol_PAW_021	pbe_US_021	pbe_US_MIT
Ecut (Ry)	90	90	90	90
K-point	4 × 4 × 1	4 × 4 × 4	5 × 5 × 5	7 × 7 × 7
U_Fe_ (eV)	3.0	4.0	4.0	4.0
U_O_ (eV)	7.0	/	/	/
Lattice constants(Å)	a = 5.046 (5.04) c = 13.743 (13.75)	5.9451 (5.94 [43])	4.354 (4.33 [48])	4.335 (4.33 [48])
Magnetic moment (Bohr mag/cell)	4.49 (4.6–4.7)	4.04 (3.59–3.76 [44])	4.0925 (3.33–4.2 [49])	4.13 (3.33–4.2 [49])
Band gaps (eV)	2.836 (2.0–2.2 [51,52,53])	0.62 (0.14 [54,55])		1.91 (2.4 [56])

**Table 3:** The bulk V_0_, bulk modulus B_0_, and first derivative of bulk modulus with respect to pressure B_p_ of three iron oxides (including FeO*) at 0 pressure under different conditions by the Birch–Murnaghan equation of state. Bold is the result closest to the experimental value.

Iron Oxide	Pseudo Potential	B_0_/GPa	B_P_	V_O_/Å^3^
Fe_2_O_3_	sol_PAW_021	201.43	0.05	303.82
	pbe_US_021	221.55	0.12	304.14
	pbe_US_MIT	**223.58**	0.16	303.03
	pbe_US_MIT (no-m)	279.47	−0.25	253.66
	pbe_US_MIT (no-U)	180.93	0.74	296.39
	pbe_US_MIT (no-m/U)	272.73	−0.44	253.49
	Pbe(U = 4) [20]	187.56		
	expt [60]	230 ± 5		
Fe_3_O_4_	sol_PAW_021	134.65	−7.88	149.14
	pbe_US_021	135.18	−5.87	153.68
	pbe_US_021 (U_O_ = 7)	**195.25**	0.25	148.63
	pbe_US_MIT	174.87	−0.34	149.96
	pbe_US_MIT (no-m)	230.48	−0.44	127.63
	pbe_US_MIT (no-U)	174.85	0.30	144.92
	pbe_US_MIT (no-m/U)	248.15	−0.26	125.38
	Pbe(U = 4) [20]	176.00		
	expt [61,62]	200 ± 25		
FeO	sol_PAW_021	138.07	−0.05	81.59
	pbe_US_021	**191.71**	−0.23	82.64
	pbe_US_MIT	200.12	−0.02	81.62
	pbe_US_MIT (no-m)	273.59	0.81	68.31
	pbe_US_MIT (no-U)	226.51	1.29	72.66
	pbe_US_MIT (no-m/U)	226.60	1.26	72.66
FeO*	sol_PAW_021	208.27	−0.17	40.11
	sol_PAW_021 (U_O_ = 8)	211.17	0.60	40.26
	pbe_US_021	**192.62**	−0.30	41.17
	pbe_US_MIT	200.90	−0.13	40.73
	pbe_US_MIT (no-m)	273.74	0.79	34.15
	pbe_US_MIT (no-U)	195.51	1.81	37.50
	pbe_US_MIT (no-m/U)	273.74	0.79	34.15
	Pbe(U = 4) [20]	166.36		
	LDA [63]	173.63		
	Pbe(U = 4) [20]	166.36		
	expt [64,65]	151–180		

## Data Availability

Not applicable.

## Supplementary Materials

The following supporting information can be downloaded at: https://www.mdpi.com/article/10.3390/ma15238316/s1, Figure S1: Fermi level of Fe_3_O_4_ in the case of sol_PAW_021, pbe_US_021, k = 5 as a function of U value. The dotted line in the figure is where the line breakpoint of the corresponding color appears.; Figure S2: Degauss result of Fe_3_O_4_; Figure S3: Degauss results of FeO; Figure S4: Regular test curve of U_O_ in Fe_3_O_4_. Different colors correspond to different U_Fe_; Figure S5. Regular test curve of U_O_ in FeO*. Different colors correspond to different U_Fe_; Table S1: Fe_2_O_3_ detailed test parameters; Table S2: Fe_3_O_4_ detailed test parameters; Table S3: FeO and FeO* detailed test parameters; Table S4: Names of pseudopotential set and pseudopotentials; Table S5: Ecut parameter results of Fe_2_O_3_; Table S6: Smeaing parameter results of Fe_2_O_3_; Table S7: K-point parameter results of Fe_2_O_3_; Table S8: U_Fe_ results of Fe_2_O_3_; Table S9: U_O_ results of Fe_2_O_3_; Table S10: Pseudopotential Results of Fe_2_O_3_; Table S11: Degauss results of Fe_2_O_3_; Table S12: Starting magnetization results of Fe_2_O_3_; Table S13: Bate results of Fe_2_O_3_; Table S14: conv_thr results of Fe_2_O_3_; Table S15: Nbnd results of Fe_2_O_3_; Table S16: K-point results of Fe_3_O_4_; Table S17: Pseudopotential results of Fe_3_O_4_; Table S18: Ecut results of Fe_3_O_4_; Table S19: U_Fe_ results of Fe_3_O_4_ when the pseudopotential is sol_PAW_021 group; Table S20: U_Fe_ results of Fe_3_O_4_ when the pseudopotential is pbe_US_021 group; Table S21: U_Fe_ results of Fe_3_O_4_ when the k-point is 5 × 5 × 5; Table S22: Degauss result of Fe_3_O_4_; Table S23: Starting magnetization result of Fe_3_O_4_; Table S24: Nbnd results of Fe_3_O_4_; Table S25: Conv_thr results for Fe_3_O_4_; Table S26: Bate results of Fe_3_O_4_; Table S27: K-point results of FeO; Table S28: Pseudopotential results of FeO; Table S29: Ecut results of FeO; Table S30: U results of FeO; Table S31: Seaming results of FeO; Table S32: Degauss results of FeO; Table S33: Starting magnetization results of FeO; Table S34: Beta results of FeO; Table S35: Nbnd results of FeO; Table S36: Conv_thr results of FeO; Table S37: Pseudopotential results of FeO*; Table S38: Ecut results of FeO*; Table S39: U results of FeO*; Table S40: K-piont results of FeO*; Table S41: Seaming results of FeO*; Table S42: Degauss results of FeO*; Table S43: Starting magnetization results of FeO*; Table S44: Beta results of FeO*; Table S45: Nbnd results of FeO*; Table S46: Conv_thr results of FeO*. Refs [11,20,30,31,32,33,38,39,40,41,42,43,44,45,46,47,48,49,54,66] are cited in Supplementary Materials.
