# Supplementary material for: Density Functional Studies on the Atomistic Structure and Properties of Iron Oxides: A Parametric Study
Source: Materials (Basel). 2022 Nov 23;15(23):8316. doi: 10.3390/ma15238316 (PMC9740064; doi:10.3390/ma15238316)
Supplement: Supplementary file 1 [file materials-15-08316-s001.zip › materials-2011951-supplementary.pdf]

materials

Supporting information

Density Functional Studies on the Atomistic Structure and Properties of Iron Oxides: A Parametric Study

This Supporting information includes:

Tables. S1 to S45

S1 to S3 is the detailed parameter selection and sequence of iron oxide parameter test.

S4 is the name of the set of the pseudopotential.

S5 ~ S15 are the detailed results of Fe_2O_3 parameter test.

S16 ~ S26 are the detailed results of Fe_3O_4 parameter test.

S27 ~ S36 are the detailed results of FeO parameter test.

S37 ~ S46 are the detailed results of FeO parameter test.

Figures. S1 to S5

Table S1. Fe_2O_3 detailed test parameters (During the calculation, only the tested parameters change, and the rest of the parameters remain unchanged. The final selected value is bolded).

<table>
<tr><td colspan="2">ecut</td><td colspan="3">50, 60, 70, 80, 90, 100</td></tr>
<tr><td colspan="2">smearing</td><td colspan="3">“mv [1]”, ”gaussian”</td></tr>
<tr><td colspan="2">k</td><td colspan="3">(3 3 1), (4 4 1), (4 4 2), (5 5 1)</td></tr>
<tr><td colspan="2">U_{Fe}/eV</td><td colspan="3">3, 3.5, 4, 4.5, 5, 5.5</td></tr>
<tr><td colspan="2">ecut/Ry (U_O = 7eV [2])</td><td colspan="3">50, 60, 70, 80, 90, 100</td></tr>
<tr><td colspan="2">Fe</td><td colspan="3">Fe.pbe-spn-rrkjus_psl.0.2.1.UPF
Fe.pbesol-spn-kjpaw_psl.0.2.1.UPF
Fe.pbesol-spn-kjpaw_psl.1.0.0.UPF
Fe.pbesol-spn-rrkjus_psl.1.0.0.UPF
Fe.pbe-sp-van_mit.UPF</td></tr>
<tr><td colspan="2">O</td><td colspan="3">O.pbe-n-rrkjus_psl.0.1.UPF
O.pbesol-n-kjpaw_psl.0.1.UPF
O.pbesol-n-kjpaw_psl.1.0.0.UPF
O.pbesol-n-rrkjus_psl.1.0.0.UPF
O.pbe-rrkjus.UPF</td></tr>
<tr><td colspan="2">degauss</td><td colspan="3">0.1, 0.05, 0.01, 0.001</td></tr>
<tr><td colspan="2">starting_magnetization(1)</td><td colspan="3">0.01, 0.1, 0.2, 0.3, 0.4, 0.5, 0.6, 0.7, 0.8, 0.9, 1</td></tr>
<tr><td colspan="2">conv_thr</td><td colspan="3">1.0d-x(x = 5, 6, 7, 8, 9, 10)</td></tr>
<tr><td colspan="2">mixing_beta</td><td colspan="3">0.1, 0.2, 0.3, 0.4, 0.5, 0.6, 0.7, 0.8, 0.9</td></tr>
<tr><td colspan="2">nbnd</td><td colspan="3">80 100 150 200 250 300 350 400 450 500 550</td></tr>
<tr><td colspan="2">Initial cell parameters/Å</td><td colspan="3">A = B = 5.035, C = 13.720,
cosAB = −0.5, cosAC = cosBC = 0 = 0</td></tr>
<tr><td rowspan="8">Atomic posittions</td><td></td><td>X</td><td>Y</td><td>Z</td></tr>
<tr><td>Fe1</td><td>0.333333343</td><td>0.666666687</td><td>0.02166667</td></tr>
<tr><td>Fe1</td><td>0.333333343</td><td>0.666666687</td><td>0.311666667</td></tr>
<tr><td>Fe1</td><td>0</td><td>0</td><td>0.355000019</td></tr>
<tr><td>Fe1</td><td>0</td><td>0</td><td>0.644999981</td></tr>
<tr><td>Fe1</td><td>0.666666687</td><td>0.333333343</td><td>0.688333333</td></tr>
<tr><td>Fe1</td><td>0.666666687</td><td>0.333333343</td><td>0.978333354</td></tr>
<tr><td>Fe2</td><td>0</td><td>0</td><td>0.144999996</td></tr>
</table>

Fe2	0.666666687	0.333333343	0.188333333
Fe2	0.666666687	0.333333343	0.478333324
Fe2	0.333333343	0.666666687	0.521666646
Fe2	0.333333343	0.666666687	0.811666667
Fe2	0	0	0.855000019
O	0.291999996	0	0.25
O	0.708000004	0	0.75
O	0	0.291999996	0.25
O	0	0.708000004	0.75
O	0.708000004	0.708000004	0.25
O	0.291999996	0.291999996	0.75
O	0.958666682	0.333333343	0.583333313
O	0.374666661	0.333333343	0.083333336
O	0.666666687	0.625333309	0.583333313
O	0.666666687	0.041333336	0.083333336
O	0.374666661	0.041333336	0.583333313
O	0.958666682	0.625333309	0.083333336
O	0.625333309	0.666666687	0.916666687
O	0.041333336	0.666666687	0.416666657
O	0.333333343	0.958666682	0.916666687
O	0.333333343	0.374666661	0.416666657
O	0.041333336	0.374666661	0.916666687
O	0.625333309	0.958666682	0.416666657

Table S2. Fe_3O_4 detailed test parameters (To make the calculation more efficient, we changed the order of the tests according to the influence importance of the parameters, so the partial order is different from Fe_2O_3.).

k	(3 3 3), **(4 4 4)**, (5 5 5) (6 6 6) (7 7 7)
Fe	Fe.pbe-spn-rrkjus_psl.0.2.1.UPF<br>**Fe.pbesol-spn-kjpaw_psl.0.2.1.UPF**<br>Fe.pbesol-spn-kjpaw_psl.1.0.0.UPF<br>Fe.pbesol-spn-rrkjus_psl.1.0.0.UPF<br>Fe.pbe-sp-van_mit.UPF
O	O.pbe-n-rrkjus_psl.0.1.UPF<br>**O.pbesol-n-kjpaw_psl.0.1.UPF**<br>O.pbesol-n-kjpaw_psl.1.0.0.UPF<br>O.pbesol-n-rrkjus_psl.1.0.0.UPF<br>O.pbe-rrkjus.UPF
ecut/Ry	75, 80, **90**, 95, 100
U_{Fe}/eV	2.5, 3, 3.5, **4**, 4.5, 5
U_{Fe}/eV (k-point is 5 × 5 × 5)	2.5, 3, 3.5, 4, 4.5, 5
U_{Fe}/eV (k-point is 5 × 5 × 5)	2.5, 3, 3.5, 4, 4.5, 5
smearing	"mv", "**gaussian**"
degauss	0.1-0.9, 0.05, **0.01**, 0.001
starting_magnetization(1)	0.01, 0.1, 0.2, 0.3, **0.4**, 0.5, 0.6, 0.7, 0.8, 0.9, 1
mixing_beta	0.1, 0.2, 0.3, 0.4, **0.5**, 0.6, 0.7, 0.8, 0.9
conv_thr	1.0d-x(x = 6, 7, 8, **9**, 10)

nbnd	50 100 150 **200** 250 300 350			
Initial cell parameters/Å	A = 5.935454, cos AB = cos AC = cos BC = 0.5			
		X	Y	Z
	Fe2	0.125	0.125	0.125
	Fe2	0.875	0.875	0.875
	Fe1	0.5	0.5	0.5
	Fe1	0.5	0.5	0
	Fe1	0.5	0	0.5
	Fe1	0	0.5	0.5
Atomic posittions	O	0.254900008	0.254900008	0.254900008
	O	0.254900008	0.254900008	0.735299945
	O	0.254900008	0.735299945	0.254900008
	O	0.735299945	0.254900008	0.254900008
	O	0.745100021	0.745100021	0.264700025
	O	0.745100021	0.745100021	0.745100021
	O	0.745100021	0.264700025	0.745100021
	O	0.264700025	0.745100021	0.745100021

Table S3. FeO and FeO* detailed test parameters. FeO final results are in bold, and FeO* final results are underlined.

k	(3 3 3), (4 4 4), **(5 5 5)** (6 6 6) (7 7 7)			
Fe	Fe.pbe-spn-rrkjus_psl.0.2.1.UPF<br>**Fe.pbesol-spn-kjpaw_psl.0.2.1.UPF**<br>Fe.pbesol-spn-kjpaw_psl.1.0.0.UPF<br>Fe.pbesol-spn-rrkjus_psl.1.0.0.UPF<br>Fe.pbe-sp-van_mit.UPF			
O	O.pbe-n-rrkjus_psl.0.1.UPF<br>**O.pbesol-n-kjpaw_psl.0.1.UPF**<br>O.pbesol-n-kjpaw_psl.1.0.0.UPF<br>O.pbesol-n-rrkjus_psl.1.0.0.UPF<br>O.pbe-rrkjus.UPF			
ecut/Ry	50 60 70 80 **90** 100 110			
U_{Fe}/eV	2.5, 3, 3.5, **4**, 4.5, 5			
smearing	**"mv"**, "gaussian"			
degauss	0.1, **0.2**-0.9, 0.05, 0.01, 0.001			
starting_magnetization(1)	**0.01**, 0.1, 0.2, 0.3, 0.4, 0.5, 0.6, 0.7, 0.8, 0.9, 1			
mixing_beta	0.1, 0.2, 0.3, 0.4, 0.5, 0.6, 0.7, **0.8**, 0.9			
conv_thr	1.0d-x(x = 6, 7, **8**, 9, 10)			
nbnd	50 70 **90** 120 150 180 200			
Initial cell parameters/Å (FeO)	A = 4.33, cosAB = cosAC = cosBC = 0			
		X	Y	Z
	Fe1	0	0	0
	Fe2	0	0.5	0.5
	Fe1	0.5	0	0.5
Atomic positions (FeO)	Fe2	0.5	0.5	0
	O	0.5	0.5	0.5
	O	0.5	0	0
	O	0	0.5	0
	O	0	0	0.5

Initial cell parameters/Å (FeO*)	A = 6.126, B = C = 3.063, cosAB = cosAC = cosBC = 0.5			
		X	Y	Z
Atomic positions (FeO*)	Fe1	0	0	0
	O	0.25	0.5	0.5
	O	0.75	0.5	0.5
	Fe2	0.5	0	0

Table S4. Names of pseudopotential set and pseudopotentials.

group name	pseudo potential of Fe	pseudo potential of O
pbe_US_021	Fe.pbe-spn-rrkjus_psl.0.2.1.UPF	O.pbe-n-rrkjus_psl.0.1.UPF
sol_PAW_021	Fe.pbesol-spn-kjpaw_psl.0.2.1.UPF	O.pbesol-n-kjpaw_psl.0.1.UPF
sol_PAW_100	Fe.pbesol-spn-kjpaw_psl.1.0.0.UPF	O.pbesol-n-kjpaw_psl.1.0.0.UPF
sol_US_021	Fe.pbesol-spn-rrkjus_psl.1.0.0.UPF	O.pbesol-n-rrkjus_psl.1.0.0.UPF
pbe_US_MIT	Fe.pbe-sp-van_mit.UPF	O.pbe-rrkjus.UPF

Table S5. Ecut parameter results of Fe_2O_3.

ecut	Time/min	a/Å	c/Å	cell volume/Å^3	Energy/eV	magnetic moment/ug	band gap/ev	fermi energy/ev
50	33m42.49s	5.0897	13.8515	2097.0347	−49734.4502	4.2542	2.1390	10.2409
60	33m 0.94s	5.1268	13.9882	2148.7159	−49735.3068	4.2750	1.9502	11.0334
70	38m21.00s	5.1165	13.9477	2133.9164	−49736.0377	4.2717	1.9500	11.2898
80	40m 9.46s	5.1219	13.9722	2142.1634	−49736.5892	4.2750	1.9547	11.1381
90	39m37.08s	5.1285	13.9936	2151.0150	−49736.7449	4.2783	1.9568	10.9943
100	46m 1.10s	5.1305	14.0007	2153.7100	−49736.7731	4.2792	1.9572	10.9489
ext [3–5]		5.04	13.75			4.6–4.7	2.0–2.2	
pbe+u(4.3)[6]		5.104	13.907			4.2	2.0	

Table S6. Smeaing parameter results of Fe_2O_3.

seaming	Time/min	a/Å	c/Å	cell volume/Å3	Energy/eV	magnetic moment/ug	band gap/ev	fermi energy/ev
mv	39m37.08s	5.1285	13.9936	2151.0150	−49736.7449	4.2783	1.9568	10.9943
gaussian	40m13.18s	5.1285	13.9936	2151.0124	−49736.7449	4.2783	1.9566	10.9944

Table S7. K-point parameter results of Fe_2O_3.

K-point	Time/min	a/Å	c/Å	cell volume/Å3	Energy/eV	magnetic moment/ug	band gap/ev	fermi energy/ev
3 3 1	39m37.08s	5.1285	13.9936	2151.0150	−49736.7449	4.2783	1.9566	10.9944
4 4 1	73m	5.1284	13.9945	2151.0520	−49736.7464	4.2783	1.9563	10.9912
4 4 2	137m	5.1284	13.9947	2151.0341	−49736.7466	4.2783	1.9563	10.9903
5 5 1	104m	5.1284	13.9946	2151.0569	−49736.7462	4.2783	1.9563	10.9907

Table S8. U_{Fe} results of Fe_2O_3.

U	Time/min	a/Å	c/Å	cell volume/Å3	Energy/eV	magnetic moment/ug	band gap/ev	fermi energy/ev
No U	1000m	5.0154	13.8810	2040.5966	−49396.4545	3.7625	0.5955	11.3446
3	83m	5.1001	13.9664	2123.1104	−49377.9151	4.1800	1.5583	10.9992
3.5	77m	5.1112	13.9770	2133.9525	−49375.1989	4.2217	1.7128	10.7709
4	72m	5.1221	13.9874	2144.6774	−49372.5712	4.2583	1.8667	11.0887
4.3	71m	5.1284	13.9945	2151.0342	−49371.0352	4.2783	1.9563	10.9911
4.5	72m	5.1325	13.9993	2155.2533	−49370.0275	4.2908	2.0127	10.9269
5	72m	5.1427	14.0117	2165.6888	−49367.5634	4.3192	2.1368	10.7693
5.5	74m	5.1525	14.0244	2175.9177	−49365.1739	4.3450	2.1839	10.6163

Table S9. U_O results of Fe_2O_3.

ecut	Time/min	a/Å	c/Å	cell volume/Å^3	Energy/eV	magnetic moment/ug	band gap/ev	fermi energy/ev
50	14m56.61s	5.0406	13.7218	2037.4841	−49710.1876	4.4442	2.3900	10.3542
60	19m16.09s	5.0667	13.7941	2069.5208	−49710.8656	4.4558	2.3790	9.7995
70	22m38.46s	5.0578	13.7611	2057.3496	−49711.5703	4.4533	2.3400	10.0213
80	25m55.26s	5.0630	13.7851	2065.1261	−49712.1670	4.4567	2.1406	9.8697
90	30m 8.61s	5.0688	13.8029	2072.5999	−49712.3458	4.4592	2.2898	9.7422
100	31m 0.83s	5.0704	13.8086	2074.7450	−49712.3662	4.4600	2.3460	9.7038

Table S10. Pseudopotential Results of Fe_2O_3.

pseudo potential	Time/min	a/Å	c/Å	magnetic moment/ug	band gap/ev
pbe_US_021	72	5.128	13.995	4.28	1.96
sol_PAW_021	83	5.066	13.808	4.23	1.85
sol_PAW_100	95	5.060	13.790	4.23	1.84
pbe_US_MIT	69	5.087	13.895	4.35	2.26
ext[3-5]		5.04	13.75	4.6–4.7	2.0–2.2
pbe+u(4.3)[6]		5.104	13.907	4.2	2.0
GGA-PW91[7]		4.997,	13.854	0.3	

B3LYP[8]	5.12	13.82	3	
PBE+U[6]	5.104	13.907	2	
HSE(a = 0.25)[6]	5.00	13.62	4.13	3.45
Pbe+u(U = 4eV)[6]	5.04	13.73	4.15	2.08

Table S11. Degauss results of Fe_2O_3.

degauss	Time/min	a/Å	c/Å	cell volume/Å^3	Energy/eV	magnetic moment/ug	band gap/ev	fermi energy/ev
0.001	72m	5.1284	13.9945	2151.0321	−49736.7466	4.2783	0.0007	10.8994
0.01	71m	5.1284	13.9945	2151.0319	−49736.7466	4.2783	1.9563	10.9912
0.05	80m	5.1286	13.9941	2151.1830	−49736.7767	4.2692	1.9559	10.7114
0.1	155m	5.1410	13.9921	2161.2337	−49738.7567	4.0892	1.9472	10.4947
0.2	convergence NOT achieved after 100 iterations: stopping							

Table S12. Starting magnetization results of Fe_2O_3.

m	Time/min	a/Å	c/Å	cell volume/Å^3	Energy/eV	magnetic moment/ug	band gap/ev	fermi energy/ev
0.01	71m	5.1284	13.9945	2151.0317	−49736.7466	4.2783	1.9564	10.9912
0.1	77m	5.1284	13.9945	2151.0342	−49736.7466	4.2783	1.9563	10.9911
0.2	76m	5.1284	13.9945	2151.0326	−49736.7466	4.2783	1.9563	10.9912
0.3	72m	5.1284	13.9945	2151.0346	−49736.7466	4.2783	1.9563	10.9912
0.4	71m	5.1284	13.9945	2151.0342	−49736.7466	4.2783	1.9563	10.9911
0.5	72m	5.1284	13.9945	2151.0340	−49736.7466	4.2783	1.9563	10.9912
0.6	75m	5.1284	13.9945	2151.0333	−49736.7466	4.2783	1.9563	10.9912
0.7	76m	5.1284	13.9945	2151.0340	−49736.7466	4.2783	1.9563	10.9912
0.8	71m	5.1284	13.9945	2151.0341	−49736.7466	4.2783	1.9563	10.9912
0.9	70m	5.1284	13.9945	2151.0341	−49736.7466	4.2783	1.9563	10.9912
1	77m	5.1284	13.9945	2151.0339	−49736.7466	4.2783	1.9563	10.9912

Table S13. Bate results of Fe_2O_3.

bate	Time/min	a/Å	c/Å	cell volume/Å^3	Energy/eV	magnetic moment/ug	fermi energy/ev
0.1	78m	5.1284	13.9945	2151.0354	−49736.7466	4.2783	10.9911
0.2	71m	5.1284	13.9945	2151.0360	−49736.7466	4.2783	10.9912
0.3	70m	5.1284	13.9945	2151.0365	−49736.7466	4.2783	10.9912
0.4	69m	5.1284	13.9945	2151.0364	−49736.7466	4.2783	10.9911
0.5	71m	5.1284	13.9945	2151.0342	−49736.7466	4.2783	10.9911
0.6	80m	5.1284	13.9945	2151.0360	−49736.7466	4.2783	10.9911
0.7	78m	5.1284	13.9945	2151.0335	−49736.7466	4.2783	10.9911
0.8	80m	5.1284	13.9945	2151.0336	−49736.7466	4.2783	10.9911
0.9	83m	5.1284	13.9945	2151.0337	−49736.7466	4.2783	10.9912

Table S14. conv_thr results of Fe_2O_3.

conv_thr	Time/min	a/Å	c/Å	cell volume/Å^3	Energy/eV	magnetic moment/ug	fermi energy/ev
1.0d-5	65m	5.1283	13.9943	2150.9425	−49736.7465	4.2783	10.9926
1.0d-6	61m	5.1283	13.9948	2151.0124	−49736.7466	4.2783	10.9908
1.0d−7	67m	5.1284	13.9945	2151.0646	−49736.7466	4.2783	10.9908
1.0d-8	69m	5.1284	13.9945	2151.0346	−49736.7466	4.2783	10.9912
1.0d-9	71m	5.1284	13.9945	2151.0342	−49736.7466	4.2783	10.9911
1.0d−10	78m	5.1284	13.9945	2151.0374	−49736.7466	4.2783	10.9911

Table S15. Fe_2O_3 nbnd results, according to the QE PW. x parameter manual, calculate the total number of external electrons in the system half of the sum is 102, so nbnd >102, and as a conductor metal, so 20%, nbnd >122.4. Tried nbnd = 80、100 and did stop counting with too few bands.

Nbnd(102)	Time/min	a/Å	c/Å	cell volume/Å^3	Energy/eV	magnetic moment/ug	band gap/ev	fermi energy/ev
150	internal error, cannot bracket Ef							
200	37m25.55s	5.1284	5.1284	2151.0342	−49736.7466	4.2783	1.9563	10.8891
228	40m59.22s	5.1284	5.1284	2151.0334	−49736.7466	4.2783	1.9563	10.3561
250	43m58.85s	5.1284	5.1284	2151.0345	−49736.7466	4.2783	1.9563	11.1217
300	53m16.86s	5.1284	5.1284	2151.0337	−49736.7466	4.2783	1.9563	10.8968
350	1h 2m	5.1284	5.1284	2151.0345	−49736.7466	4.2783	1.9563	10.8123
400	1h12m	5.1284	5.1284	2151.0343	−49736.7466	4.2783	1.9563	10.9911
450	1h32m	5.1284	5.1284	2151.0322	−49736.7466	4.2783	1.9563	10.9943
500	1h37m	5.1284	5.1284	2151.0319	−49736.7466	4.2783	1.9563	10.6825
550	1h51m	5.1284	5.1284	2151.0367	−49736.7466	4.2783	1.9563	10.6565

Table S16. k-point results of Fe_3O_4.

K-point	Time/min	a/Å	cell volume/Å3	Energy/eV	magnetic moment/ug	fermi energy/ev
3	13m36.79s	6.0449	1054.0163	−24411.4135	4.09	10.5590
4	23m44.88s	6.0448	1053.9568	−24411.4176	4.09	10.5596
5	43m11.21s	6.0448	1053.9663	−24411.4174	4.09	10.5588
6	1h 6m	6.0448	1053.9629	−24411.4174	4.09	10.5590
7	1h27m	6.0448	1053.9591	−24411.4174	4.09	10.5591

Table S17. Pseudopotential results of Fe_3O_4.

pseudo potential	Time/min	a/Å	magnetic moment/ug	band gap/ev
pbe_US_021	32	6.005	4.03	0.8
sol_PAW_021	32	5.945	4.04	1.05
sol_PAW_100	58	5.899	4.04	1.1
pbe_US_MIT	29	5.986	4.15	0.4

ext	5.94[9]	3.59-3.76[10]	1.92 - 2.87 [11]
LSD[12]		A:3.46-3.48, B:3.39-3.57	0
LSD+SIC		A:4.00-4.08, B:3.83-3.97	0.35
LDA+U[13](3.6 eV)		B:3.39-3.90	0.2
LDA+U[14](4eV)		B:3.48-3.94	0.18

Table S18. Ecut results of Fe_3O_4.

ecut	Time/min	a/Å	cell volume/Å^3	Energy/eV	magnetic moment/ug	band gap/ev	fermi energy/ev
75	22m 4.26s	5.9321	996.0997	−31143.5144	4.0333	1.0200	12.2367
80	24m55.00s	5.9376	998.8679	−31143.6222	4.0367	0.0700	12.1922
85	33m 0.55s	5.9422	1001.2277	−31143.6771	4.0383	1.6350	12.1537
90	32m21.48s	5.9451	1002.6540	−31143.6990	4.0400	1.0520	12.1303
95	38m15.39s	5.9463	1003.2673	−31143.7060	4.0400	1.6340	12.1203
100	46m16.97s	5.9461	1003.1901	−31143.7096	4.0400	1.6360	12.1216
ext		5.94[9]			3.59-3.76[10]	0.14[15]	

Table S19. U_{Fe} results of Fe_3O_4 when the pseudopotential is sol_PAW_021 group.

U	Time/min	a/Å	cell volume/Å^3	Energy/eV	magnetic moment/ug	band gap/ev	fermi energy/ev	Time/min
2.5	29m44.35s	5.9159	60.0000	987.9571	−31148.4444	3.9483	0.5	12.0787
3	34m 6.91s	5.9257	60.0000	992.8928	−31146.8349	3.9817	0.9	12.0947
3.5	54m30.12s	5.9426	59.9445	998.9079	−31145.2813	4.0083	1.05	12.0196
3.7	30m48.43s	5.9393	60.0000	999.7324	−31144.6286	4.0233		12.1189
3.9	28m40.52s	5.9431	60.0000	1001.6776	−31144.0077	4.0333		12.1265
4	33m30.99s	5.9451	60.0000	1002.6564	−31143.6990	4.0400	1.21	12.1303
4.2	30m 8.49s	5.9699	60.0001	1015.2924	−31143.7431	4.0583		11.1273
4.4	73m	5.9941	59.7871	1017.8113	−31143.4838	4.0550		10.8022
4.5	27m57.38s	5.9757	60.0001	1018.2210	−31142.9362	4.0717	1.22	11.0385
5	25m54.96s	5.9851	60.0002	1023.0606	−31141.6151	4.0933	1.3	10.8868

Table S20. U_{Fe} results of Fe_3O_4 when the pseudopotential is pbe_US_021 group.

U	Time/min	a/Å	α/°	cell volume/Å^3	Energy/eV	magnetic moment/ug	fermi energy/ev
2.5	29m47.56s	5.9959	60.0000	1028.5855	−24415.2588	4.0017	11.6019
3	38m 2.85s	6.0054	60.0000	1033.5170	−24413.6956	4.0317	11.6175
3.5	34m24.23s	6.0149	60.0000	1038.4088	−24412.1586	4.0600	11.6362
3.6	37m24.98s	6.0168	60.0000	1039.4054	−24411.9386	4.0550	11.5489
3.7	39m21.55s	6.0188	60.0000	1040.4180	−24411.6425	4.0600	11.5455
3.8	38m21.49s	6.0208	60.0000	1041.4529	−24411.3475	4.0667	11.5419
3.9	25m28.83s	6.0429	60.0000	1052.9893	−24411.6822	4.0850	10.5891
4	24m 2.25s	6.0448	60.0000	1053.9568	−24411.4176	4.0900	10.5596
4.5	25m43.80s	6.0540	60.0000	1058.7724	−24410.1121	4.1100	10.4089
5	28m26.02s	6.0631	60.0001	1063.5779	−24408.8357	4.1283	10.2513

Table S21. U_{Fe} results of Fe_3O_4 when the k-point is 5×5×5.

U	Time/min	a/Å	α/°	cell volume/Å^3	Energy/eV	magnetic moment/ug	fermi energy/ev
2.5	43m26.88s	5.9161	60.000	988.0897	−31148.4453	3.950	12.064
3	51m 4.42s	5.9257	60.000	992.9131	−31146.8362	3.982	12.086
3.5	35m27.84s	5.9354	60.000	997.7697	−31145.2548	4.012	12.104
4	51m 8.16s	5.9451	60.000	1002.6503	−31143.7001	4.040	12.123
4.5	108m	5.9953	59.800	1019.0249	−31143.2269	4.060	10.758
5	40m10.85s	5.9851	60.000	1023.0634	−31141.6149	4.093	10.886

Table S22. Degauss result of Fe_3O_4.

degauss	Time/min	a/Å	cell volume/Å^3	Energy/eV	magnetic moment/ug	fermi energy/ev
0.0001	convergence NOT achieved after 100 iterations: stopping					
0.001	31m22.82s	5.9444	1002.3027	−31143.7699	4.0300	12.0857
0.01	29m27.66s	5.9451	1002.6550	−31143.6990	4.0400	12.1303
0.02	49m20.19s	5.9491	1004.7195	−31143.8040	4.0567	12.0727
0.04	39m22.69s	5.9627	1011.6106	−31144.2054	4.1117	11.7908
0.05	convergence NOT achieved after 100 iterations: stopping					
0.06	convergence NOT achieved after 100 iterations: stopping					
0.08	27m52.28s	5.9701	1015.3892	−31145.5283	3.9917	11.1349
0.1	29m56.98s	5.9721	1016.3724	−31146.2967	3.9483	11.1428
0.2	30m 3.40s	5.9735	1017.1279	-31155.4485	3.1117	10.9999
0.3	26m36.87s	5.9967	1028.9846	-31175.5845	0.0000	10.7917

Table S23. Starting magnetization result of Fe_3O_4.

m	Time/min	a/Å	cell volume/Å^3	Energy/eV	magnetic moment/ug	fermi energy/ev
0.01	43m34.01s	5.9451	1002.6553	−31143.6990	4.0400	12.1303
0.1	34m11.22s	5.9451	1002.6550	−31143.6990	4.0400	12.1303
0.2	36m28.97s	5.9451	1002.6559	−31143.6990	4.0400	12.1303
0.3	convergence NOT achieved after 100 iterations: stopping					
0.4	29m54.96s	5.9451	1002.6551	−31143.6990	4.0400	12.1303
0.5	29m12.24s	5.9451	1002.6566	−31143.6990	4.0400	12.1303
0.6	33m27.24s	5.9451	1002.6568	−31143.6990	4.0400	12.1303
0.7	30m49.46s	5.9451	1002.6559	−31143.6990	4.0400	12.1303
0.8	35m43.03s	5.9451	1002.6566	−31143.6990	4.0400	12.1303
0.9	37m19.10s	5.9451	1002.6569	−31143.6990	4.0400	12.1303
1	35m33.42s	5.9451	1002.6574	−31143.6990	4.0400	12.1302

Table S24. nbnd results of Fe_3O_4.

nbnd	Time/min	a/Å	cell volume/Å^3	Energy/eV	magnetic moment/ug	fermi energy/ev
50	Too few band					
100	21m27.57s	5.9451	1002.6544	−31143.6990	4.04	12.1303
150	27m42.29s	5.9451	1002.6568	−31143.6990	4.04	12.1303
200	31m 2.35s	5.9451	1002.6576	−31143.6990	4.04	12.1302
250	50m41.34s	5.9451	1002.6577	−31143.6990	4.04	12.1302
300	52m35.28s	5.9451	1002.6557	−31143.6990	4.04	12.1303
350	61m	5.9451	1002.6564	−31143.6990	4.04	12.1303

Table S25. conv_thr results for Fe_3O_4.

c	Time/min	a/Å	cell volume/Å^3	Energy/eV	magnetic moment/ug	fermi energy/ev
6	21m44.24s	5.9444	1002.3631	−31143.6990	4.0400	12.1352
7	23m12.04s	5.9450	1002.6181	−31143.6990	4.0400	12.1309
8	28m16.76s	5.9451	1002.6639	−31143.6990	4.0400	12.1301
9	34m 1.98s	5.9451	1002.6551	−31143.6990	4.0400	12.1303
10	34m 5.46s	5.9451	1002.6568	−31143.6990	4.0400	12.1303
11	39m28.06s	5.9451	1002.6566	−31143.6990	4.0400	12.1303

Table S26. bate results of Fe_3O_4.

beta	Time/min	a/Å	cell volume/Å^3	Energy/eV	magnetic moment/ug	fermi energy/ev
0.1	convergence NOT achieved after 100 iterations: stopping					
0.2	27m51.01s	5.9661	1013.3221	−31144.2872	4.0500	11.1858
0.3	33m17.85s	5.9661	1013.3210	−31144.2872	4.0500	11.1858
0.4	29m12.34s	5.9661	1013.3208	−31144.2872	4.0500	11.1858
0.5	30m37.59s	5.9451	1002.6568	−31143.6990	4.0400	12.1303
0.6	29m21.40s	5.9661	1013.3210	−31144.2872	4.0500	11.1858
0.7	32m24.71s	5.9451	1002.6562	−31143.6990	4.0400	12.1303
0.8	34m 5.28s	5.9451	1002.6575	−31143.6990	4.0400	12.1302
0.9	convergence NOT achieved after 100 iterations: stopping					

Table S27. K-point results of FeO.

K-point	Time/min	a/Å	b/Å	c/Å	cell volume/Å^3	Energy/eV	magnetic moment/ug	fermi energy/ev	a/b
3	2m 0.40s	4.3566	4.3562	4.3566	557.9567	−15661.1592	4.0850	13.8761	1.0001
4	4m43.12s	4.3563	4.3562	4.3563	557.8913	−15661.1522	4.0875	13.8740	1.0000
5	4m30.78s	4.3562	4.3561	4.3562	557.8455	−15661.1530	4.0900	13.8753	1.0000
6	9m46.69s	4.3563	4.3561	4.3563	557.8528	−15661.1530	4.0900	13.8750	1.0000
7	10m14.95s	4.3563	4.3561	4.3563	557.8575	−15661.1530	4.0900	13.8749	1.0000

Table S28. Pseudo potential results of FeO.

pseudo potential	time	a	b	magnetic moment (ug)	a/b
pbe_US_MIT	5m32.33s	4.34	4.34	4.1425	1.0017
pbe_US_021	4m37.66s	4.36	4.36	4.0900	1.0000
sol_PAW_021	5m12.71s	4.30	4.30	4.0525	1.0002
sol_PAW_100	5m 7.09s	4.30	4.30	4.0500	1.0002
ext		4.33 [16]		3.33–4.2 [17]	
GGA+PBE		4.268		3.59	
PBE+U(5eV)[18]		4.3333		3.74	
LDA[19]		4.18		3.44	
B3PW91[20]		4.35		4.15	

Table S29. Ecut results of FeO.

ecut	Time/min	a/Å	b/Å	cell volume/Å^3	Energy/eV	magnetic moment/ug	fermi energy/ev	a/b
50	2m33.47s	4.3315	4.3314	548.4218	−15660.5277	4.0725	14.2078	1.0000
60	4m 3.41s	4.3512	4.3509	555.8805	−15660.7331	4.0825	13.9395	1.0001

70	4m28.05s	4.3437	4.3434	553.0338	−15660.9509	4.0825	14.0435	1.0001
80	4m30.08s	4.3506	4.3503	555.6693	−15661.1070	4.0850	13.9519	1.0001
90	4m37.03s	4.3562	4.3561	557.8455	−15661.1530	4.0900	13.8753	1.0000
100	5m15.85s	4.3574	4.3573	558.2985	−15661.1610	4.0900	13.8594	1.0000
110	5m30.13s	4.3567	4.3565	558.0073	−15661.1698	4.0900	13.8698	1.0000
ext		4.33[16]				3.33-4.2[17]		

Table S30. U results of FeO.

U	Time/min	a/Å	b/Å	cell volume/Å^3	Energy/eV	magnetic moment/ug	fermi energy/ev	a/b
2.5	3m52.13s	4.3290	4.3277	547.2948	−15664.1730	4.0625	14.1177	1.0003
3	3m59.37s	4.3394	4.3386	551.3059	−15663.2922	4.0900	14.0109	1.0002
3.5	4m19.25s	4.3475	4.3471	554.4517	−15662.4085	4.0950	13.9378	1.0001
4	4m45.06s	4.3539	4.3537	556.9591	−15661.5142	4.0925	13.8898	1.0000
4.5	4m51.24s	4.3595	4.3590	559.0427	−15660.6077	4.0850	13.8582	1.0001
5	convergence NOT achieved after 100 iterations: stopping							

Table S31. seaming results of FeO.

seaming	Time/min	a/Å	b/Å	cell volume/Å^3	Energy/eV	magnetic moment/ug	fermi energy/ev	a/b
gauss	6m44.16s	4.4138	4.4144	580.3683	−15669.1784	3.3100	13.0350	0.9999
mv	4m45.06s	4.3539	4.3537	556.9591	−15661.5142	4.0925	13.8898	1.0000

Table S32. Degauss results of FeO.

d	Time/min	a/Å	b/Å	cell volume/Å^3	Energy/eV	magnetic moment/ug	fermi energy/ev	a/b
0.0001	10m36.46s	4.5614	4.1995	589.6557	−15663.0754	3.8125	12.8115	1.0862
0.001	13m14.26s	4.5615	4.1995	589.6637	−15663.0756	3.8125	12.8114	1.0862
0.01	7m39.23s	4.6018	4.1695	595.8487	−15663.9024	3.8100	12.2769	1.1037
0.05	21m17.10s	4.3358	4.4989	570.7316	−15660.4085	3.8625	13.7901	0.9637
0.1	15m50.98s	4.3747	4.4619	576.2578	−15661.2906	3.9575	13.5665	0.9804
0.2	4m41.48s	4.3539	4.3537	556.9591	−15661.5142	4.0925	13.8898	1.0000
0.3	7m11.10s	4.2900	4.2767	531.1667	−15659.1891	3.9875	14.5424	1.0031
0.4	9m 0.45s	4.1868	4.1843	494.9608	−15658.7065	1.5800	15.7530	1.0006

0.5	6m52.11s	4.4103	4.4103	578.8876	−15663.0887	0.0000	13.3311	1.0000

Table S33. Starting magnetization results of FeO.

m	Time/min	a/Å	b/Å	cell volume/Å^3	Energy/eV	magnetic moment/ug	fermi energy/ev	a/b
0.01	4m56.48s	4.3540	4.3538	556.9707	−15661.5142	4.0925	13.8893	1.0000
0.1	4m34.35s	4.3539	4.3538	556.9671	−15661.5142	4.0925	13.8895	1.0000
0.2	4m25.00s	4.3540	4.3538	556.9703	−15661.5142	4.0925	13.8894	1.0000
0.3	4m40.06s	4.3539	4.3537	556.9580	−15661.5142	4.0925	13.8898	1.0000
0.4	4m23.23s	4.3539	4.3538	556.9632	−15661.5142	4.0925	13.8896	1.0000
0.5	4m23.88s	4.3540	4.3538	556.9713	−15661.5142	4.0925	13.8893	1.0000
0.6	4m35.36s	4.3539	4.3538	556.9658	−15661.5142	4.0925	13.8895	1.0000
0.7	4m41.55s	4.3539	4.3537	556.9433	−15661.5142	4.0925	13.8903	1.0000
0.8	4m45.33s	4.3540	4.3538	556.9713	−15661.5142	4.0925	13.8893	1.0000
0.9	4m39.72s	4.3540	4.3538	556.9774	−15661.5142	4.0925	13.8891	1.0000
1	4m47.52s	4.3540	4.3538	556.9785	−15661.5142	4.0925	13.8891	1.0000

Table S34. Beta results of FeO.

beta	Time/min	a/Å	b/Å	cell volume/Å^3	Energy/eV	magnetic moment/ug	fermi energy/ev	a/b
0.1	4m41.49s	4.3540	4.3538	556.9952	−15661.5142	4.0925	13.8885	1.0001
0.2	4m48.77s	4.3541	4.3538	557.0076	−15661.5142	4.0925	13.8880	1.0001
0.3	4m56.43s	4.3540	4.3537	556.9717	−15661.5142	4.0925	13.8893	1.0001
0.4	4m56.77s	4.3540	4.3538	556.9728	−15661.5142	4.0925	13.8893	1.0000
0.5	4m36.85s	4.3540	4.3538	556.9664	−15661.5142	4.0925	13.8895	1.0000
0.6	4m37.23s	4.3540	4.3538	556.9729	−15661.5142	4.0925	13.8893	1.0000
0.7	4m26.15s	4.3540	4.3538	556.9752	−15661.5142	4.0925	13.8892	1.0000
0.8	4m35.55s	4.3539	4.3537	556.9580	−15661.5142	4.0925	13.8898	1.0000
0.9	4m29.91s	4.3540	4.3538	556.9711	−15661.5142	4.0925	13.8893	1.0000

Table S35. nbnd results of FeO.

nbnd	Time/min	a/Å	b/Å	cell volume/ Å³	Energy/eV	magnetic moment/ug	fermi energy/ev	a/b
40	too few bands							
50	6m 6.20s	4.3450	4.3454	553.6063	−15661.3360	4.0900	14.0116	0.9999
70	3m47.58s	4.3540	4.3538	556.9792	−15661.5142	4.0925	13.8890	1.0000
90	4m26.39s	4.3540	4.3538	556.9692	−15661.5142	4.0925	13.8894	1.0000
120	5m20.50s	4.3539	4.3538	556.9658	−15661.5142	4.0925	13.8895	1.0000
150	6m19.39s	4.3539	4.3538	556.9580	−15661.5142	4.0925	13.8898	1.0000

Table S36. conv_thr results of FeO.

conv_thr	Time/min	a/Å	b/Å	cell volume/ Å³	Energy/eV	magnetic moment/ug	fermi energy/ev	a/b
6	4m12.92s	4.3534	4.3538	556.8386	−15661.5141	4.0925	13.8941	0.9999
7	4m29.68s	4.3538	4.3535	556.8862	−15661.5141	4.0925	13.8923	1.0001
8	4m52.36s	4.3539	4.3537	556.9580	−15661.5142	4.0925	13.8898	1.0000
9	5m15.00s	4.3539	4.3538	556.9670	−15661.5142	4.0925	13.8895	1.0000
10	5m27.13s	4.3540	4.3538	556.9710	−15661.5142	4.0925	13.8893	1.0000
11	5m34.45s	4.3540	4.3538	556.9722	−15661.5142	4.0925	13.8893	1.0000

Table S37. Pseudo potential results of FeO*.

pseudo potential	time	a/Å	β/°	magnetic moment (ug)
pbe_US_MIT	10m53.02s	4.335	59.94	4.130
pbe_US_021	11m18.12s	4.355	59.83	4.065
sol_PAW_021	12m45.82s	4.301	59.813	4.030
sol_PAW_100	12m0.60s	4.297	59.82	4.025
ext		4.33 [16]		3.33–4.2 [17]
GGA+PBE		4.268		3.59
PBE+U(5eV)[18]		4.333		3.74
LDA[19]		4.18		3.44
B3PW91[20]		4.35		4.15

Table S38. Ecut results of FeO*.

ecut	Time/min	a/Å	β/°	cell volume/Å³	Energy/eV	magnetic moment/ug	fermi energy/ev	band gap/ev
70	10m58.36s	4.335	59.944	274.7822	−7755.8275	4.125	14.1720	1.9
80	18m39.07s	4.335	59.9436	274.7881	−7755.8293	4.125	14.1715	1.85
90	13m18.32s	4.335	59.9438	274.8826	−7755.8300	4.130	14.1645	1.91
100	15m42.99s	4.3349	59.9453	274.8492	−7755.8299	4.130	14.1668	1.9

110	16m20.92s	4.3349	59.9465	274.8550	−7755.8302	4.130	14.1663	1.91
120	17m52.57s	4.3346	59.9429	274.7905	−7755.8305	4.130	14.1711	1.9
ext		4.33[16]				3.33-4.2[17]		

Table S39. U results of FeO*.

U	Time/min	a/Å	β/°	cell volume/Å^3	Energy/eV	magnetic moment/ug	fermi energy/ev	band gap/ev
3	16m 4.84s	4.3242	59.8855	272.8266	−7756.6247	4.135	14.1966	1.77
3.5	15m56.41s	4.3301	59.9164	273.9467	−7756.2308	4.135	14.1715	1.81
4	13m42.52s	4.3351	59.9438	274.8826	−7755.8300	4.130	14.1645	1.91
4.5	11m49.49s	4.3387	59.9843	275.5794	−7755.4229	4.120	14.1804	1.99
5	12m19.40s	4.3427	59.9937	276.3483	−7755.0110	4.120	14.1981	2.1

Table S40. K-piont results of FeO*.

K-point	Time/min	a/Å	β/°	cell volume/Å^3	Energy/eV
3	1m52.79s	4.3358	59.8130	275.0203	−7755.7866
4	3m47.22s	4.3351	59.9302	274.8856	−7755.8260
5	6m12.11s	4.3346	59.9429	274.3039	−7755.8297
6	10m41.97s	4.3349	59.9452	274.8453	−7755.8300
7	13m14.92s	4.3351	59.9438	274.8826	−7755.8300

Table S41. seaming results of FeO*.

seaming	Time/min	a/Å	β/°	cell volume/Å3	Energy/eV	magnetic moment/ug	fermi energy/ev
gauss	21m44.63s	4.4150	59.9332	290.3762	−7834.5800	3.2850	13.0161
mv	13m18.32s	4.3350	59.9438	274.8826	−7755.8300	4.1300	14.1645

Table S42. Degauss results of FeO*.

d	Time/min	a/Å	β/°	cell volume/Å^3	Energy/eV	magnetic moment/ug	fermi energy/ev
0.0001	convergence NOT achieved after 100 iterations: stopping 4.1995 589.6557 −15663.0754 3.8125 12.8115						
0.001	26m39.56s	4.3426	61.2247	275.7483	−7755.5007	3.785	14.0698
0.01	36m24.17s	4.3683	60.1383	281.2358	−7756.6831	3.785	13.1567

0.05	22m25.14s	4.3871	59.0617	284.5751	−7756.5985	3.815	12.6692
0.1	28m56.21s	4.3597	60.8652	279.3036	−7755.7243	3.820	14.3445
0.2	13m 7.96s	4.3351	59.9438	274.8826	−7755.8300	4.130	14.1645
0.3	24m45.93s	4.0545	60	224.8854	−7752.3154	0	17.2399
0.4	22m20.34s	4.1142	60	234.9733	−7753.3626	0	16.5802
0.5	16m14.09s	4.3705	60	281.6835	−7755.4591	0	13.6828

Table S43. Starting magnetization results of FeO*.

m	Time/min	a/Å	β/°	cell volume/$Å^3$	Energy/eV	magnetic moment/ug	fermi energy/e v
0.01	15m31.66s	4.3349	59.943 0	274.8526	−7755.8300	4.13	14.1667
0.1	15m18.89s	4.3349	59.944 3	274.8528	−7755.8300	4.13	14.1667
0.2	19m 6.58s	4.3349	59.944 4	274.8503	−7755.8300	4.13	14.1668
0.3	15m 1.07s	4.3348	59.946 6	274.8358	−7755.8300	4.13	14.1679
0.4	15m10.26s	4.3348	59.948 3	274.8369	−7755.8300	4.13	14.1678
0.5	15m 0.06s	4.33496	59.942 7	274.8646	−7755.8300	4.13	14.1658
0.6	14m32.44s	4.3349	59.944 2	274.8451	−7755.8300	4.13	14.1672
0.7	18m55.84s	4.3348	59.943 6	274.8273	−7755.8300	4.13	14.1685
0.8	14m19.66s	4.3352	59.943 4	274.9098	−7755.8300	4.13	14.1625
0.9	12m56.95s	4.3349	59.960 3	274.8585	−7755.8300	4.13	14.1661
1	14m44.98s	4.3349	59.943 2	274.8599	−7755.8300	4.13	14.1662

Table S44. Beta results of FeO*.

beta	Time/min	a/Å	β/°	cell volume/$Å^3$	Energy/eV	magnetic moment/ug	fermi energy/e v
0.1	14m37.25s	4.3347	59.9440	274.8150	−7755.8300	4.13	14.1695
0.2	12m10.57s	4.3348	59.9455	274.8342	−7755.8300	4.13	14.1680
0.3	10m 1.51s	4.3351	59.9476	274.8935	−7755.8300	4.13	14.1636
0.4	10m17.79s	4.3352	59.9514	274.9129	−7755.8300	4.13	14.1622
0.5	11m51.24s	4.3347	59.9445	274.8083	−7755.8300	4.13	14.1699
0.6	10m29.63s	4.3351	59.9554	274.8839	−7755.8300	4.13	14.1643
0.7	11m25.82s	4.3347	59.9449	274.8113	−7755.8300	4.13	14.1697
0.8	12m15.44s	4.3349	59.9440	274.8481	−7755.8300	4.13	14.1670

0.9	10m47.83s	4.3349	59.9451	274.8591	−7755.8300	4.13	14.1662

Table S45. nbnd results of FeO*.

nbnd	Time/min	a/Å	β/°	cell volume/Å^3	Energy/eV	magnetic moment/ug	fermi energy/ev
35	9m58.41s	4.33499	59.9468	274.87047	−7755.8300	4.13	14.1653
40	9m48.39s	4.33479	59.942	274.83226	−7755.8300	4.13	14.1682
50	12m52.50s	4.3349	59.9440	274.8481	−7755.8300	4.13	14.1670
70	15m26.68s	4.3348	59.9466	274.8358	−7755.8300	4.13	14.1679
90	19m46.27s	4.3349	59.9432	274.8490	−7755.8300	4.13	14.1669

Table S46. conv_thr results of FeO*.

conv_thr	Time/min	a/Å	β/°	cell volume/Å^3	Energy/eV	magnetic moment/ug	fermi energy/ev
6	12m16.56s	4.3350	59.9454	556.8386	−15661.5141	4.0925	13.8941
7	12m41.38s	4.3350	59.9379	556.8862	−15661.5141	4.0925	13.8923
8	12m44.29s	4.3349	59.9440	556.9580	−15661.5142	4.0925	13.8898
9	12m34.91s	4.3349	59.9450	556.9670	−15661.5142	4.0925	13.8895
10	13m44.32s	4.3349	59.9449	556.9710	−15661.5142	4.0925	13.8893
11	14m34.09s	4.3349	59.9449	556.9722	−15661.5142	4.0925	13.8893

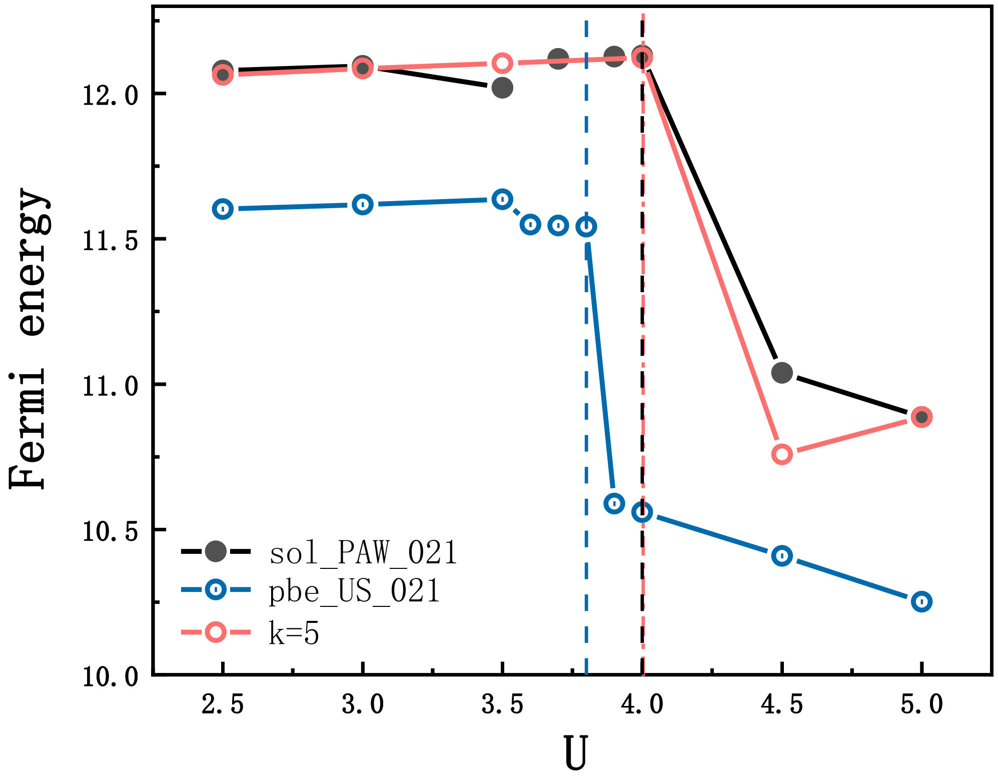

Figure S1. Fermi level of Fe_3O_4 in the case of sol_PAW_021, pbe_US_021, k = 5 as a function of U value. The dotted line in the figure is where the line breakpoint of the corresponding color appears.

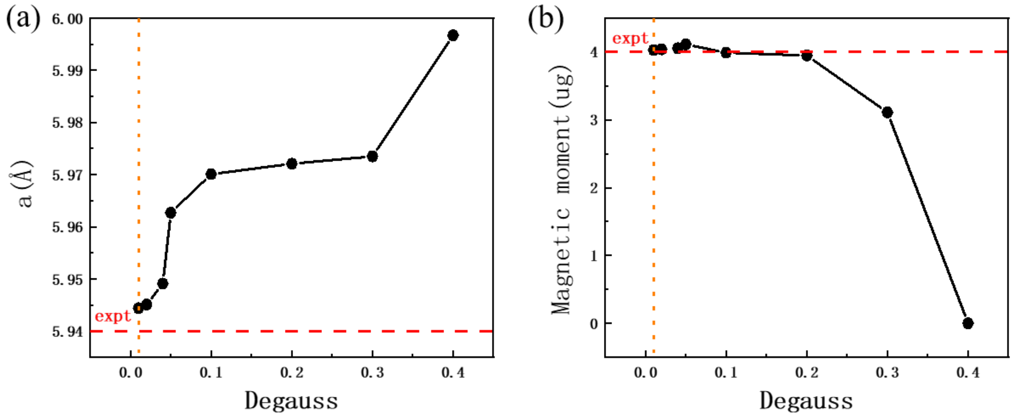

Figure S2. Degauss result of Fe_3O_4. (**a**) is the change of cell parameters with Degauss, and (**b**) is the change of magnetic moment with Degauss. The orange dotted line is the position of the final value, and the red line is the experimental value.

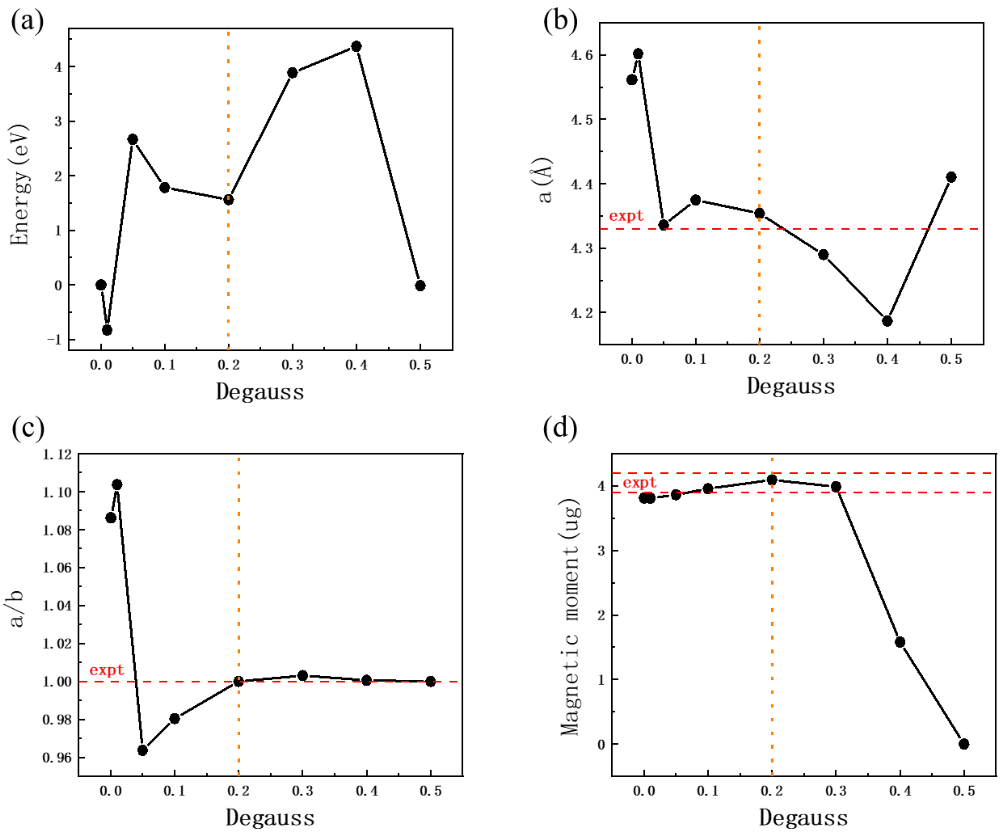

Figure S3. Degauss results of FeO. (**a**) is the change of energy with Degauss, (**b**) is the change of cell parameters with Degauss, (c) is the change of a/b with Degauss, and (d) is the change of magnetic moment with Degauss. The orange dotted line is the position of the final value, and the red line is the experimental value.

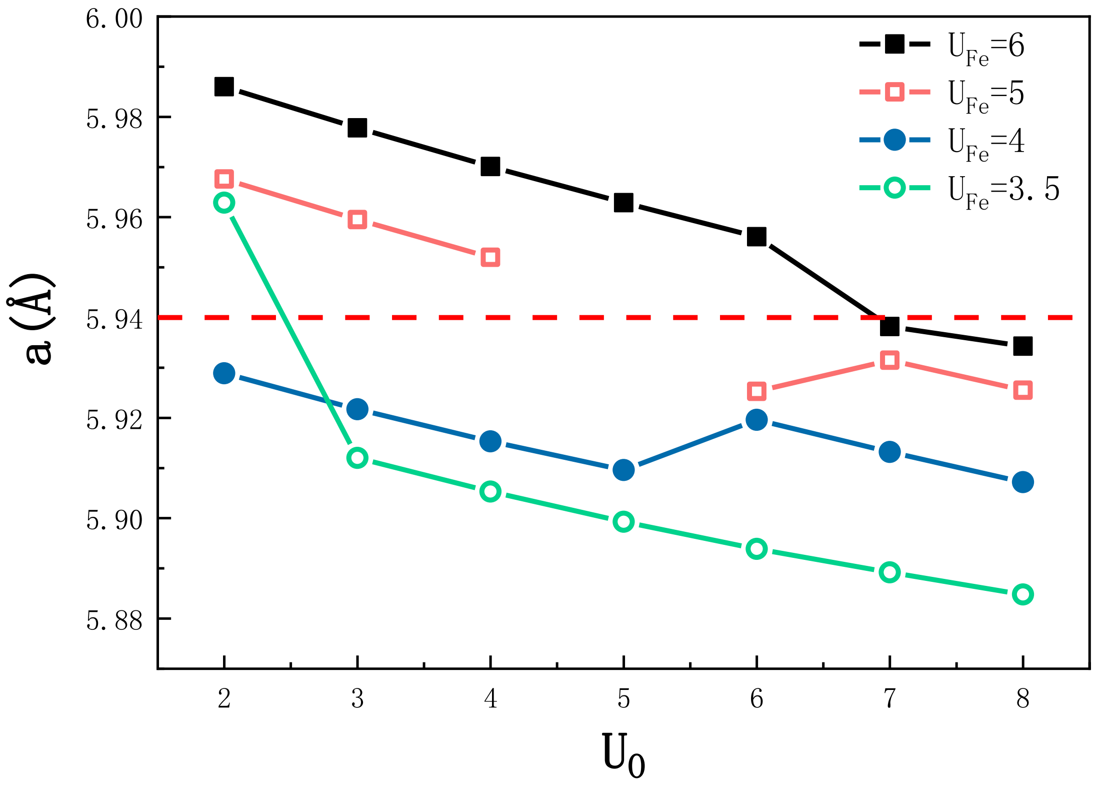

Figure S4. Regular test curve of U_O in Fe_3O_4. Different colors correspond to different U_{Fe}.

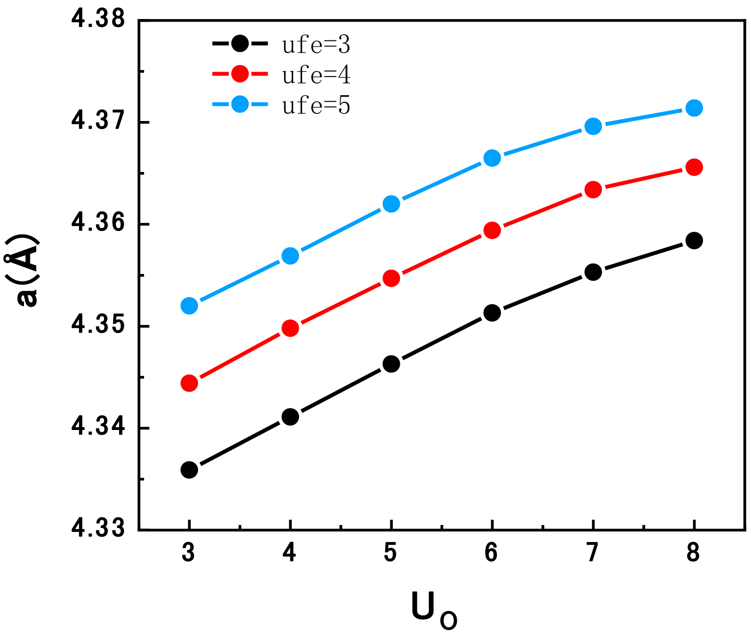

Figure S5. Regular test curve of U_O in FeO*. Different colors correspond to different U_{Fe}.

References

1. Marzari, N.; Vanderbilt, D.; Vita, A.D.; Payne, M.C. Thermal Contraction and Disordering of the Al (110) Surface. *Physical Review Letters* **1999**, *82*.
2. Shousha, S.; Khalil, S.; Youssef, M. A complete ab initio thermodynamic and kinetic catalogue of the

defect chemistry of hematite α-Fe2O3, its cation diffusion, and sample donor dopants. *Physical Chemistry Chemical Physics* **2021**, *23*, 25518–25532, doi:10.1039/D1CP03394H.
3. Cox, D.E.; Takei, W.J.; Miller, R.C.; Shirane, G. A magnetic and neutron diffraction study of the Fe2O3-V2O3 system. *Journal of Physics & Chemistry of Solids* **1963**, *23*, 863–874.
4. Krén, E.; Szabó, P.; Konczos, G. Neutron diffraction studies on the (1-x) Fe2O3 - xRh2O3 system. *Physics Letters* **1965**, *19*, 103–104.
5. Finger, L.W.; Hazen, R.M. Crystal structure and isothermal compression of Fe2O3, Cr2O3, and V2O3 to 50 kbars. *Journal of Applied Physics* **1980**, *51*, 5362–5367.
6. Meng, Y.; Liu, X.W.; Huo, C.; Guo, W.P.; Cao, D.B.; Peng, Q.; Dearden, A.; Gonze, X.; Yang, Y.; Wang, J. When Density Functional Approximations Meet Iron Oxides. *Journal of Chemical Theory & Computation* **2016**, 5132.
7. Rollmann, G.; Rohrbach, A.; Entel, P.; Hafner, J. First-principles calculation of the structure and magnetic phases of hematite. *Physical Review B* **2004**, *69*.
8. Wilson, N.C.; Russo, S.P. Hybrid density functional theory study of the high-pressure polymorphs of Fe2O3 hematite. *Physical Review B* **2009**, *79*, 094113.
9. Wright, J.P.; Attfield, J.P.; Radaelli, P.G. Charge ordered structure of magnetite Fe3O4below the Verwey transition. *Physical Review B* **2002**.
10. Rakhecha; Murthy. Spin-transfer due to covalency for the tetrahedral-site Fe3+ions in Fe3O4. *Journal of Physics C Solid State Physics* **1978**.
11. Saha, S.; Jana, M.; Khanra, P.; Samanta, P.; Koo, H.; Murmu, N.C.; Kuila, T. Band gap modified boron doped NiO/Fe3O4 nanostructure as the positive electrode for high energy asymmetric supercapacitors. *Rsc Advances* **2015**, *6*.
12. Szotek, Z.; Temmerman, W.M.; Svane, A.; Petit, L.; Stocks, G.M.; Winter, H. Ab initio study of charge order in Fe3O4. *Physical Review B* **2003**, *68*.
13. Horng-Tay, J.; Y, G.G.; J, H.D. Charge-orbital ordering and Verwey transition in magnetite. *Physical review letters* **2004**, *93*.
14. I, L.; N, Y.A.; N, A.V.; A, K.M.; I, A.V. Charge and orbital order in Fe3O4. *Physical review letters* **2004**, *93*.
15. Noh, J.; Osman, O.I.; Aziz, S.G.; Winget, P.; Brédas, J. A density functional theory investigation of the electronic structure and spin moments of magnetite. *Science and Technology of Advanced Materials* **2014**, *15*, 044202.
16. Pasternak, M.P.; Pasternak, M.P.; Taylor, R.D.; Jeanloz, R.; Li, X.; Nguyen, J.H.; Mccammon, C. High Pressure Collapse of Magnetism in Fe0.94O: Mssbauer Spectroscopy Beyond 100 GPa. *Physical Review Letters* **1997**, *79*, 5046–5049.
17. Roth, W.L. Magnetic Structures of MnO, FeO, CoO, and NiO. *Physical Review* **1958**, *110*, 1333–1341.
18. Zhang, W.-B.; Deng, Y.-H.; Hu, Y.-L.; Han, K.-L.; Tang, B.-Y. Structural distortion of B 1 -structured MnO and FeO. *Solid State Communications* **2007**, *142*.
19. Tran, F.; Blaha, P.; Schwarz, K.; Novak, P. Hybrid exchange-correlation energy functionals for strongly correlated electrons: Applications to transition-metal monoxides. *Physical review. B, Condensed Matter And Materials Physics* **2006**, *74*, p.155108.155101−155108.155110.
20. Alfredsson, M.; David Price, G.; Catlow, C.R.A.; Parker, S.C.; Orlando, R.; Brodholt, J.P. Electronic structure of the antiferromagnetic B1-structured FeO. *Physical Review B* **2004**, *70*, 165111,

doi:10.1103/PhysRevB.70.165111.
